# Thermoresponsive artificial light-harvesting system with temperature-gated cascade energy transfer and photocatalysis

**DOI:** 10.1039/d5sc07946b

**Published:** 2026-02-17

**Authors:** Zhiying Wu, Menglian Hu, Guangping Sun, Tangxin Xiao

**Affiliations:** a School of Petrochemical Engineering, Changzhou University Changzhou 213164 China xiaotangxin@cczu.edu.cn; b School of Chemistry and Chemical Engineering, Nantong University Nantong 226019 China

## Abstract

Natural photosynthesis elegantly coordinates energy transfer, energy conversion, and environmental responsiveness within complex supramolecular frameworks. Replicating this multifunctionality in artificial systems remains a formidable challenge. In this work, we report a biomimetic light-harvesting system (LHS) constructed through the self-assembly of an amphiphilic molecule, TPE-CSO, which integrates aggregation-induced emission (AIE)-active tetraphenylethylene and cyanostilbene chromophores with oligo(ethylene glycol) segments. The resulting nanostructures act as efficient energy donors and exhibit thermoresponsive disassembly behavior above their lower critical solution temperature (LCST). By co-assembling TPE-CSO with two fluorescent acceptor dyes, Rh6G and SR101, we established a cascade Förster resonance energy transfer (FRET) network that boosts reactive oxygen species (ROS) generation in a temperature-dependent manner. This adaptive LHS functions as a thermosensitive photocatalyst, efficiently promoting the oxidative cleavage of 1,1-diphenylethylene derivatives with yields reaching 93%. Notably, the catalytic performance diminishes at elevated temperatures, mimicking the temperature vulnerability observed in natural photosynthetic systems. This multifunctional system represents a significant step toward biomimetic light-harvesting platforms with built-in environmental adaptability.

## Abstract 2

A supramolecular light-harvesting system (LHS) self-assembled from TPE-CSO in water enables cascade FRET, thermoresponsive ROS generation, and temperature-regulated photocatalysis, mimicking the thermal vulnerability of natural photosynthesis.

## Graphical abstract

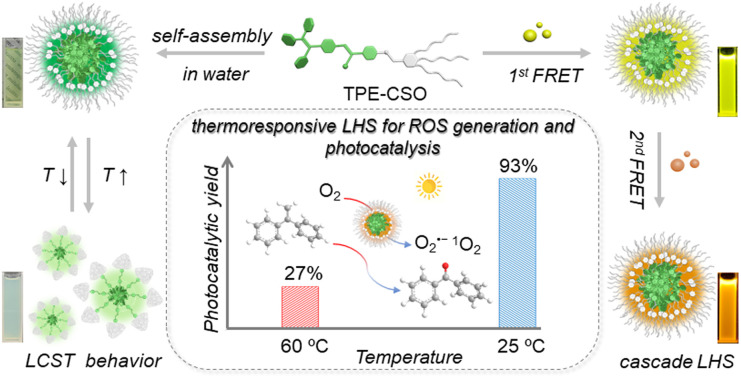

## Introduction

Photosynthesis plays a pivotal role in the efficient capture of solar energy and its subsequent conversion into chemical energy, a fundamental process underpinning life on earth.^1,2^ Among the various environmental factors affecting photosynthesis, rising global temperatures, driven by climate change, have emerged as a critical abiotic stressor.^3^ Photosynthesis is particularly vulnerable to thermal stress, often being one of the earliest physiological processes disrupted in plant cells.^4^ Numerous crop modeling studies have forecasted marked declines in agricultural productivity in elevated temperature scenarios.^5,6^ In this context, the construction of thermoresponsive artificial photosynthetic systems offers a valuable platform for elucidating the structural and functional principles of natural photosynthesis, while also advancing the development of adaptive, bioinspired energy conversion materials. While prior efforts in artificial light-harvesting systems (LHSs)^7–15^ have replicated specific aspects of natural systems, such as efficient excitation energy transfer,^16–23^ photocatalytic function,^24–29^ or thermal adaptability,^30–34^ there remains a lack of integrated systems capable of performing multistep energy funneling and subsequent chemical transformation, all while exhibiting a controlled response to temperature variations.

**Fig. 1:**
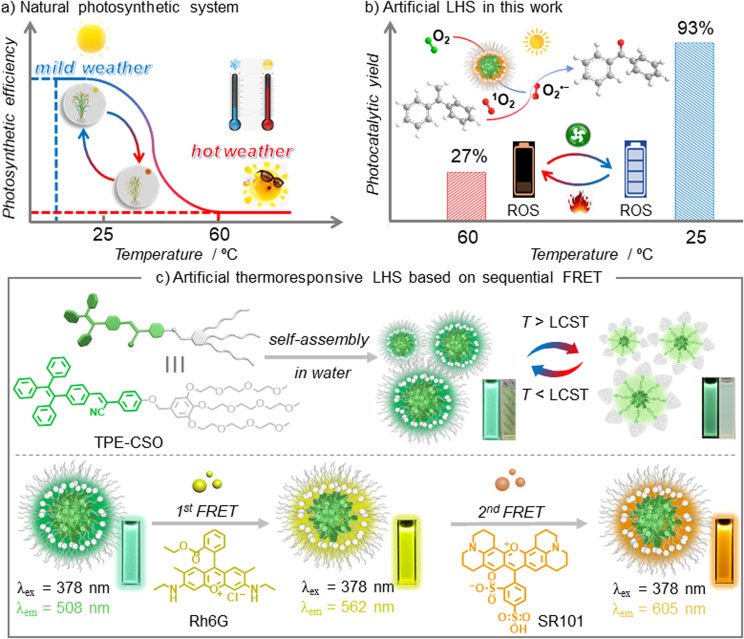
Schematic illustration of (a) the thermal response dynamics in natural photosynthetic systems; (b) temperature-gated photocatalytic switching in the artificial thermoresponsive light-harvesting system (LHS); and (c) the chemical structure of TPE-CSO and its self-assembly into a sequential LHS exhibiting thermoresponsive behavior.

Supramolecular self-assembly offers a promising approach to constructing comprehensive bionic systems.^35–38^ Ajayaghosh and co-workers reported temperature-gated cascade energy transfer in organic media, providing important conceptual guidance for the present study.^39,40^ By designing amphiphilic molecules containing chromophores, these molecules can self-assemble into nanostructures in aqueous environments, forming a light-harvesting nanoplatform.^41–44^ Incorporating aggregation-induced emission^45–49^ (AIE)-active chromophores enhances light absorption and emission within these nanoassemblies.^50–52^ Furthermore, by loading energy-matched fluorescent dyes, sequential energy transfer systems can be established *via* Förster resonance energy transfer (FRET) between chromophores.^53^ Inspired by the thermal regulation observed in natural photosynthesis, where elevated temperatures deactivate enzymes and reduce photosynthetic efficiency^54^ (Fig. 1a), we propose using oligo(ethylene glycol)^55–57^ (OEG) chains as the hydrophilic segment of amphiphilic molecules. Given their lower critical solution temperature^58–61^ (LCST) behavior, OEG chains become hydrophobic at elevated temperatures, leading to the disassembly of the nanostructures and the interruption of the light-harvesting process.

In our previous work, we reported a one-step energy-transfer LHS based on cyanostilbene, which enabled thermosensitive white-light emission.^62^ Building on this foundation, we sought to enhance the photophysical performance of the antenna donor in the assembled state by covalently integrating a cyanostilbene^63–65^ unit with a tetraphenylethylene^66–68^ chromophore. The resulting conjugated system was designed to emulate the multifunctionality of natural photosynthetic assemblies, simultaneously supporting thermosensitive light harvesting and photocatalysis (Fig. 1b). To this end, we designed and synthesized a monomeric molecule, TPE-CSO, which functions both as a supramolecular building block and as a light-harvesting antenna (Fig. 1c). TPE-CSO comprises chromophoric moieties forming the hydrophobic domain and OEG chains as the hydrophilic segment. Owing to its amphiphilic structure and AIE characteristics, TPE-CSO self-assembles into highly emissive nanoparticles (NPs) in aqueous solution. These NP suspensions are transparent at ambient temperature but become turbid upon heating, accompanied by a marked decrease in fluorescence. By co-assembling TPE-CSO NPs (donor) with commercially available dyes rhodamine 6G (Rh6G, the first acceptor) and sulforhodamine 101 (SR101, the second acceptor), we established a sequential energy-transfer LHS. This system not only enables tunable emission spanning cyan to orange *via* yellow, but also retains thermoresponsive behavior. Moreover, the sequential LHS operates as a thermoresponsive photosensitizer, promoting the efficient generation of reactive oxygen species (ROS).^69^ These ROS catalyze the oxidative cleavage of 1,1-diphenylethylene derivatives with high yields under mild conditions, whereas the catalytic activity diminishes significantly at elevated temperatures.

## Results and discussion

**Fig. 2:**
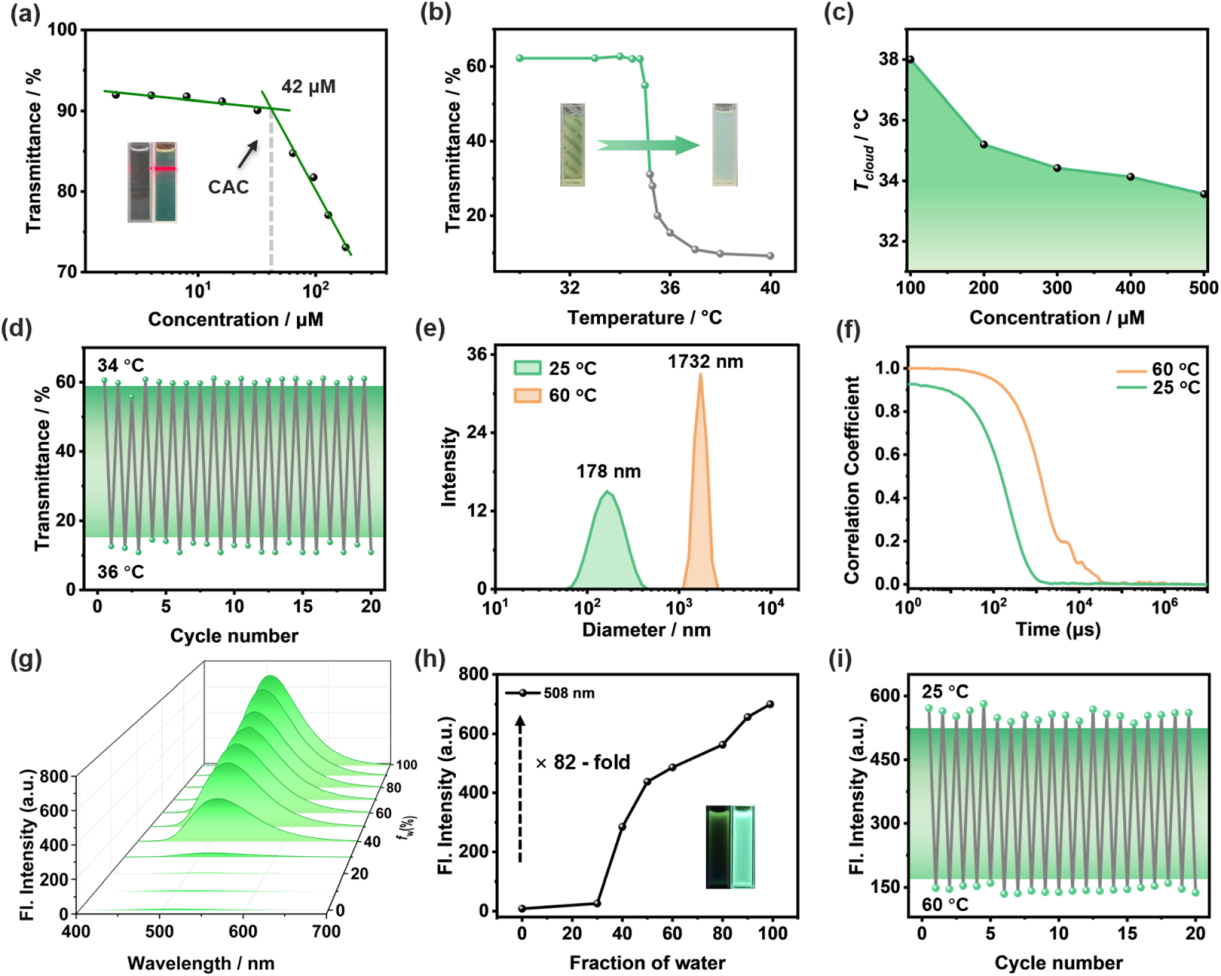
Self-assembly and thermoresponsive photophysical properties of TPE-CSO in water. (a) Transmittance of TPE-CSO at 450 nm as a function of concentration, with insets showing the Tyndall effect at 10 µM (left) and 200 µM (right). (b) Temperature-dependent transmittance of TPE-CSO aqueous solution ([c] = 200 µM), with insets showing the solution states below (left) and above (right) the cloud point temperature (*T*_cloud_). (c) Concentration-temperature phase diagram of TPE-CSO. (d) Reversible transmittance changes of TPE-CSO upon thermal cycling between 34 °C and 36 °C. (e) DLS data of TPE-CSO ([c] = 100 µM) obtained at 25 °C and 60 °C, respectively. (f) DLS correlation decay profile with time for an aqueous solution of TPE-CSO below (green) and above (red) the LCST, respectively. (g) Fluorescence (Fl.) spectra of TPE-CSO in DMSO/H_2_O mixtures with different water fractions (*f*_w_), *λ*_ex_ = 378 nm. (h) Fluorescence intensity of TPE-CSO at 508 nm as a function of the water fraction, with insets showing fluorescence images of TPE-CSO in pure DMSO (left) and pure H_2_O (right) ([c] = 100 µM). (i) Reversible fluorescence intensity changes of TPE-CSO at 508 nm upon thermal cycling between 25 °C and 60 °C.

The neutral amphiphilic monomer TPE-CSO was synthesized *via* a straightforward procedure (Scheme S1, SI) and thoroughly characterized using ^1^H NMR, ^13^C NMR, and high-resolution ESI-MS (Fig. S1–S3, SI). The self-assembly behavior of TPE-CSO in aqueous solution was then systematically examined. The optical transmittance of TPE-CSO solutions across the full spectral range was measured at varying concentrations from 2 to 256 µM (Fig. S4, SI). A clear inverse relationship was observed: as the concentration increased, transmittance progressively declined. To quantitatively assess this trend, transmittance values at 450 nm were plotted as a function of concentration (Fig. 2a). A linear fitting analysis identified a sharp inflection point at 42 µM, marking the critical aggregation concentration (CAC). Below the CAC, transmittance exhibited a gradual decrease, whereas above the CAC, a pronounced drop occurred, indicating extensive molecular self-assembly into nanostructured aggregates that significantly scatter light. This conclusion was further supported by the presence of a strong Tyndall effect in the 200 µM solution (Fig. 2a, inset right), whereas no such effect was observed at 10 µM (Fig. 2a, inset left), reinforcing the idea that aggregation occurs predominantly beyond the CAC.

The OEG chains in TPE-CSO confer both amphiphilicity and LCST responsiveness. To explore its thermal behavior, we examined the LCST phenomenon in a 200 µM aqueous solution. At room temperature, the solution remained transparent (Fig. 2b, inset), but as the temperature rose to ∼36 °C, it became turbid, reverting to transparency upon cooling. Optical transmittance measurements identified the critical transition temperature (*T*_cloud_) at 35.3 °C (Fig. 2b). The LCST behavior arises from a hydrophilic-to-hydrophobic transition in the OEG chains. Below the threshold, these chains form hydrogen bonds with water, retaining solubility. However, above the LCST, hydrogen bonds gradually break, leading to hydrophobic interactions and molecular aggregation, causing turbidity. Notably, *T*_cloud_ is concentration-dependent: increasing the concentration from 100 µM to 500 µM lowers *T*_cloud_ from 38.0 °C to 33.5 °C, reflecting enhanced hydrophobic interactions at higher concentrations (Fig. S5, SI). To illustrate this trend, we plotted a concentration–temperature distribution curve (Fig. 2c), providing crucial insight into the tunability of TPE-CSO's thermal response. Furthermore, the phase transition is highly reversible, enduring over 20 heating–cooling cycles without significant degradation (Fig. 2d), demonstrating the excellent thermal stability of TPE-CSO.

To further elucidate the morphology and size of the resulting nanostructures, dynamic light scattering (DLS), scanning electron microscopy (SEM) and transmission electron microscopy (TEM) were employed. DLS measurements show that TPE-CSO assemblies prepared at 25 °C possess an average hydrodynamic diameter (*D*_h_) of 178 nm (Fig. 2e). Consistently, SEM and TEM images reveal the formation of irregular spherical aggregates with sizes of approximately 100–200 nm (Fig. S6d and S7a, SI), in good agreement with the DLS results and thus validating the size and morphology of the self-assembled TPE-CSO nanostructures at room temperature. Upon heating above the cloud point (*T*_cloud_), DLS analysis indicates a pronounced growth in particle size accompanied by enhanced light scattering and turbidity. At 60 °C, the mean hydrodynamic diameter increases by nearly one order of magnitude to 1732 nm (Fig. 2e). In addition, the intensity autocorrelation functions of TPE-CSO display a sigmoidal decay profile at both temperatures, suggesting the existence of spherical assemblies below and above the LCST (Fig. 2f).^61^ Notably, the markedly prolonged correlation time observed at 60 °C reflects the formation of much larger aggregates. The emergence of larger particles with sigmoidal autocorrelation behavior above the LCST indicates agglomeration of dehydrated assemblies into spherical structures driven by temperature-induced water expulsion, which strongly scatter incident light and render the solution nearly opaque. By contrast, SEM images of TPE-CSO prepared at 60 °C show microscale, randomly aggregated morphologies (Fig. S7b, SI). This discrepancy likely arises because the highly adhesive, solvent-free state of the samples is not well suited for electron microscopy, but it nevertheless supports the disassembly of nanoparticles and subsequent agglomeration at elevated temperature.

To further explore the AIE characteristics of TPE-CSO, we analyzed the fluorescence behavior in DMSO/H_2_O mixed solvents (Fig. 2g). In DMSO, a good solvent, TPE-CSO remains molecularly dispersed, exhibiting negligible fluorescence (Fig. 2h, inset left). In contrast, in pure water, where self-assembly occurs, TPE-CSO aggregates into nanoparticles, emitting strong green fluorescence (Fig. 2h, inset right). This fluorescence enhancement results from the restriction of intramolecular motion, which suppresses non-radiative decay and improves quantum efficiency. Fluorescence intensity increases significantly with rising water content (*f*_w_), reaching an 82-fold enhancement in pure H_2_O compared to DMSO (Fig. 2h), confirming its robust AIE activity. Interestingly, the fluorescence emission is highly temperature-sensitive. As the temperature increases from 25 °C to 60 °C, fluorescence intensity gradually declines (Fig. S8b, SI). Below *T*_cloud_, the self-assembled NPs retain a well-packed structure, limiting molecular motion and promoting fluorescence. However, at temperatures above *T*_cloud_, the NPs loosen, increasing molecular freedom and inducing fluorescence quenching. At 60 °C, fluorescence intensity drops by 79% relative to its initial value (Fig. S8a, SI), demonstrating its high sensitivity to thermal fluctuations. To evaluate the feasibility of TPE-CSO as a fluorescent temperature sensor, we performed 20 heating–cooling cycles between 25 °C and 60 °C. The fluorescence intensity remained stable (Fig. 2i), highlighting its excellent optical reversibility and thermal durability.

**Fig. 3:**
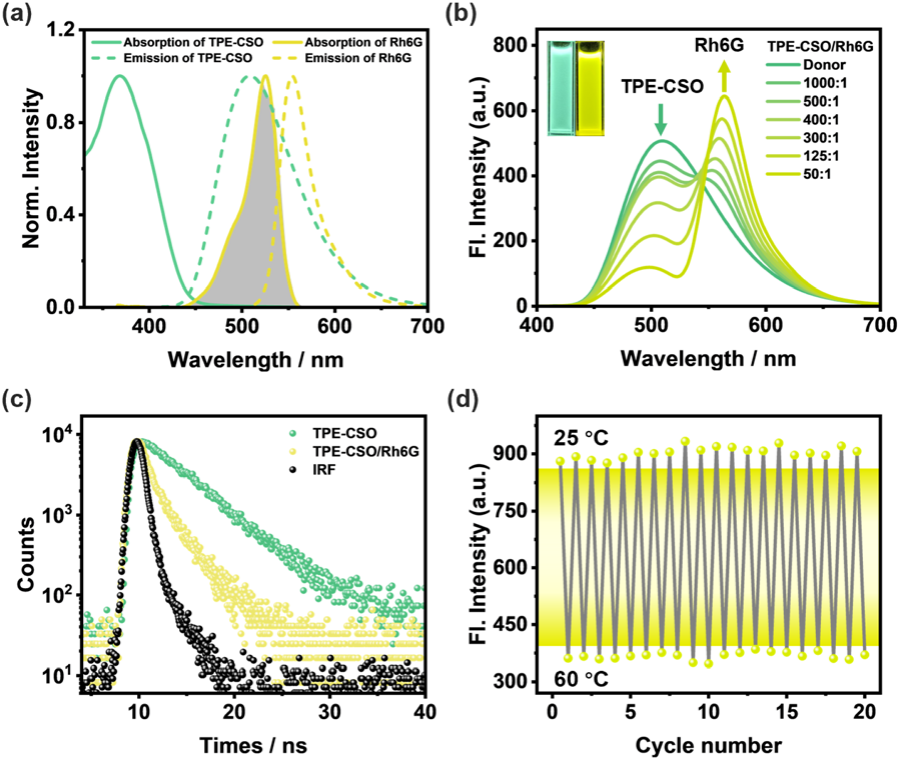
The first-step FRET. (a) Normalized fluorescence spectra (dashed curves) of TPE-CSO (green trace) and Rh6G (yellow trace), and their normalized absorption spectra (solid curves). (b) Fluorescence spectra of TPE-CSO upon incremental addition of Rh6G; inset: fluorescence images of TPE-CSO (left) and TPE-CSO/Rh6G (right), *λ*_ex_ = 378 nm. (c) Fluorescence decay profile of TPE-CSO and TPE-CSO/Rh6G (D/A1 = 50/1) monitored at 508 nm. (d) Reversible fluorescence intensity changes of TPE-CSO/Rh6G at 562 nm under thermal cycling between 25 °C and 60 °C. *λ*_ex_ = 378 nm, [TPE-CSO] = 1.0 × 10^−4^ M, and [Rh6G] = 2.0 × 10^−6^ M.

Given the photophysical behavior of TPE-CSO micellar assemblies, we propose their suitability as model systems for artificial LHSs. The spherical morphology of these micelles bears a strong resemblance to the structural organization of natural light-harvesting protein complexes, thereby offering a biomimetic platform. Furthermore, the integration of OEG side chains introduces temperature responsiveness, providing an additional dimension of tunability that more closely replicates the dynamic regulation found in natural photosynthetic systems. With this in mind, we aim to construct a thermoresponsive LHS capable of executing a two-step sequential energy transfer. As an initial step, we established a one-step FRET-based LHS by employing TPE-CSO as the energy donor (D) and Rh6G as the first acceptor (A1), chosen for their complementary spectral overlap (Fig. 3a). TEM and DLS analyses confirmed the formation of spherical NPs (TPE-CSO/Rh6G), with similar diameters upon Rh6G encapsulation (Fig. S6b and S6e, SI).

Upon gradual addition of Rh6G to aqueous TPE-CSO dispersions, the donor emission at 508 nm decreased systematically, while the emission from Rh6G at 562 nm intensified significantly (Fig. 3b and S9a, SI). This evolution in the emission profile, accompanied by a visible shift in fluorescence color from green to yellow (Fig. 3b, inset), is indicative of an efficient FRET process. To quantify the performance of the LHS, the energy transfer efficiency (*Φ*_ET_) was determined, representing the fraction of excitation energy successfully transferred from TPE-CSO to Rh6G. A gradual enhancement in *Φ*_ET_ was observed with the increasing Rh6G concentration, reaching up to 78.3% at a donor-to-acceptor ratio of 50 : 1 (Fig. S10 and Table S3, SI). This trend highlights the effective role of Rh6G as an energy acceptor and the overall robustness of the one-step FRET system. Time-resolved fluorescence measurements were further carried out for TPE-CSO and the TPE-CSO/Rh6G system, both showing biexponential decay behavior (Fig. 3c). When monitored at 508 nm, TPE-CSO displayed an average lifetime of *τ* = 5.25 ns (Table S1, SI). After introducing Rh6G at a D/A1 ratio of 50/1, the value was shortened to *τ* = 2.00 ns, confirming efficient excitation energy transfer from TPE-CSO to Rh6G. The corresponding energy transfer efficiency derived from lifetime quenching (*Φ*_ET_ = 1 − *τ*_DA_/*τ*_D_) is 62%. This value is slightly lower than that obtained from steady-state spectral quenching (78%), a difference that can be attributed to the susceptibility of the latter to artifacts such as inner-filter effects, which are often non-negligible in densely packed nanoparticle systems. Collectively, these results provide compelling evidence for the efficient and controlled establishment of a single-step energy-transfer-based artificial LHS.

The thermoresponsive behavior of the single-step LHS was systematically examined. Upon elevating the temperature from 25 °C to 60 °C, a progressive decline in the emission intensity of TPE-CSO/Rh6G at 562 nm was observed (Fig. S8d, SI). Notably, at 60 °C, the fluorescence signal at this wavelength decreased by approximately 66% relative to its initial intensity at room temperature (Fig. S8c, SI), accompanied by visible dimming in fluorescence emission (Fig. S8c, inset). From an energy transfer standpoint, the observed quenching can be attributed to a temperature-induced transition in the physicochemical properties of the TPE-CSO nanoparticles. Specifically, increased thermal input prompts a shift from hydrophilic to hydrophobic interactions within the micellar structure, leading to disordered hydrophobic aggregation. This aggregation reduces the AIE efficiency of the donor molecules and simultaneously disrupts optimal spatial proximity between the donor (TPE-CSO) and acceptor (Rh6G). As the donor–acceptor separation exceeds the Förster critical distance (∼10 nm), the FRET efficiency drops significantly, resulting in the attenuated Rh6G emission at 562 nm. Importantly, the thermoresponsive fluorescence modulation of the TPE-CSO/Rh6G NPs exhibits excellent reversibility. As illustrated in Fig. 3d, the emission intensity at 562 nm remains consistent after 20 consecutive heating–cooling cycles, underscoring the structural stability and resilience of the system. These findings collectively confirm the successful construction of a robust, thermally reversible LHS capable of efficient single-step energy transfer.

**Fig. 4:**
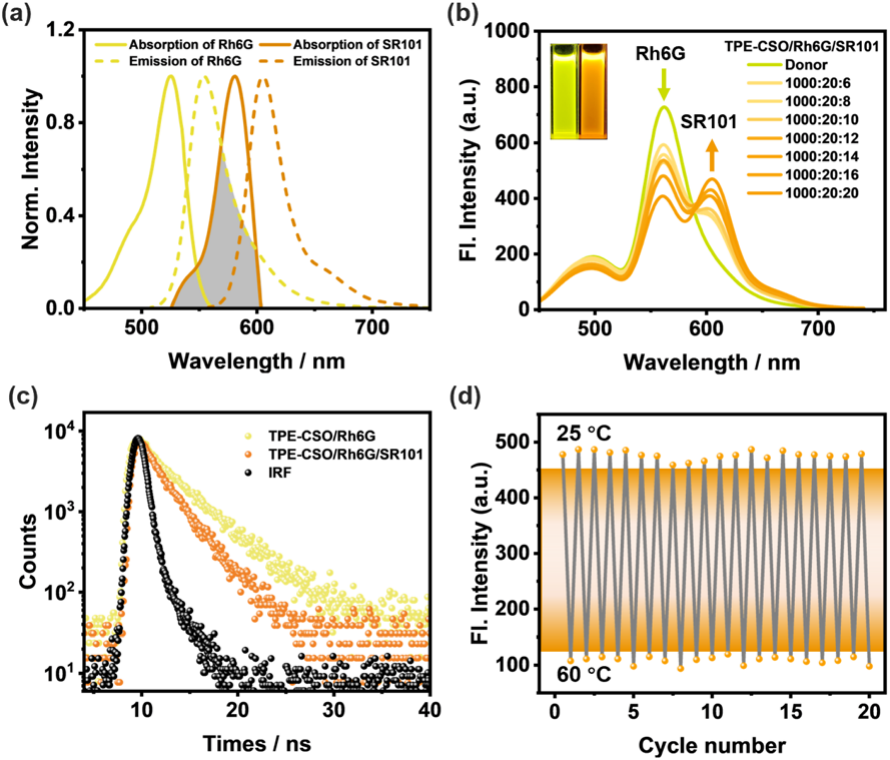
The second-step FRET. (a) Normalized fluorescence spectrum (dashed curves) of TPE-CSO/Rh6G (yellow trace) and SR101 (red trace) and their normalized absorption spectra (solid curves). (b) Fluorescence spectra of TPE-CSO/Rh6G with stepwise loading of SR101; inset: fluorescence images of TPE-CSO/Rh6G (left) and TPE-CSO/Rh6G/SR101 (right), *λ*_ex_ = 378 nm. (c) Fluorescence decay profiles of TPE-CSO/Rh6G (D/A1 = 50/1) and TPE-CSO/Rh6G/SR101 (D/A1/A2 = 1000/20/20) monitored at 562 nm. (d) Reversible fluorescence intensity changes of TPE-CSO/Rh6G/SR101 at 605 nm during thermal cycling between 25 °C and 60 °C. *λ*_ex_ = 378 nm, [TPE-CSO] = 1.0 × 10^−4^ M, [Rh6G] = 2.0 × 10^−6^ M, and [SR101] = 2.0 × 10^−6^ M.

Owing to the substantial spectral overlap between the emission of Rh6G and the absorption of SR101 (Fig. 4a), SR101 was selected as a secondary energy acceptor (A2) to facilitate sequential energy transfer from the relay acceptor Rh6G. This enabled the construction of a thermoresponsive LHS featuring cascade FRET. TEM and DLS analyses of SR101-loaded NPs revealed a slight increase in particle size and uniform size distribution, confirming the stable incorporation of SR101 (Fig. S6c and 6f, SI). Upon stepwise addition of SR101, a gradual decrease in Rh6G emission at 562 nm was observed, accompanied by a corresponding enhancement in SR101 emission at 605 nm (Fig. 4b and S9b, SI). The concurrent shift in fluorescence color from yellow to orange-red (Fig. 4b, inset) further supports the occurrence of efficient energy transfer from Rh6G to SR101.

The second-step *Φ*_ET_, defined as the fraction of excitation energy transferred from TPE-CSO/Rh6G to SR101, was subsequently calculated. *Φ*_ET_ increased steadily with higher SR101 concentrations, reaching 44.0% at a D/A1/A2 ratio of 1000/20/20 (Fig. S11 and Table S4, SI), confirming the high efficiency of the second-step energy transfer and the suitability of SR101 as an effective terminal acceptor in the cascade LHS. The evolution of fluorescence lifetimes in the second step was further investigated (Fig. 4c). When detected at 562 nm, the TPE-CSO/Rh6G system exhibited a lifetime of *τ* = 4.24 ns (Table S2, SI). Upon introduction of SR101 at a D/A1/A2 = 1000/20/20, the lifetime shortened to *τ* = 2.68 ns, demonstrating effective excitation energy transfer from Rh6G to SR101. The corresponding energy transfer efficiency calculated from the lifetime data is 37%.

The thermal responsiveness of the system was further evaluated. As shown in Fig. S8f (SI), increasing the temperature from 25 °C to 60 °C led to a pronounced decrease (82%) in the fluorescence intensity at 605 nm (Fig. S8e, SI). This quenching behavior can be attributed to the thermally induced hydrophilic-to-hydrophobic transition of TPE-CSO, which promotes non-radiative decay pathways due to disrupted packing and less efficient sequential energy transfer. Importantly, the system demonstrated excellent reversibility: during repeated heating–cooling cycles, both the emission intensity at 605 nm and the fluorescence color remained stable and tunable (Fig. 4d), highlighting the good thermal cycling durability of the cascade LHS. These findings confirm the successful development of a thermoresponsive LHS capable of two-step sequential energy transfer, offering valuable guidance for the rational design of smart, light-responsive supramolecular systems.

**Fig. 5:**
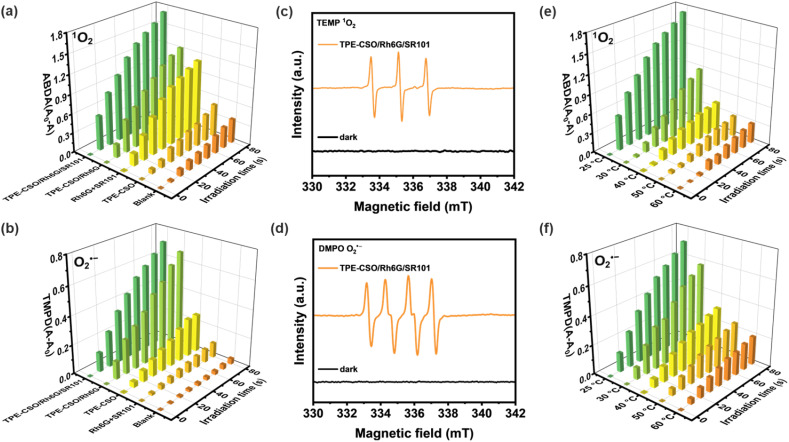
Evaluation of temperature-dependent ROS generation. Histogram of (a) ΔAbs (*A*_0_ − *A*) for ABDA at 371 nm and (b) ΔAbs (*A* − *A*_0_) for TMPD at 560 nm upon light irradiation (365–375 nm, 10 W) for different times in the presence of TPE-CSO/Rh6G/SR101, TPE-CSO/Rh6G, Rh6G + SR101 mixture, and TPE-CSO (blank: ABDA or TMPD without any additive). EPR spectra to detect (c) ^1^O_2_ in water and (d) O_2_˙^−^ in methanol using TPE-CSO/Rh6G/SR101 under dark and light conditions, using TEMP and DMPO as spin trappers, respectively. Histograms of temperature-dependent (e) ΔAbs (*A*_0_ − *A*) for ABDA at 371 nm and (f) ΔAbs (*A* − *A*_0_) for TMPD at 560 nm upon light irradiation for different times in the presence of TPE-CSO/Rh6G/SR101.

ROS play crucial roles in detoxification, therapeutic applications, and pollutant degradation due to their high reactivity.^70–72^ They are generally classified into type I (radical species, *e.g.*, O_2_˙^−^) and type II (non-radical species, *e.g.*, ^1^O_2_).^73^ To evaluate the ROS generation efficiency of the thermoresponsive LHS with sequential FRET, the overall ROS production was initially assessed using the commercially available fluorescent probe 2,7-dichlorodihydrofluorescein (DCFH).^74,75^ As shown in Fig. S12 (SI), upon 80 s of light irradiation, the fluorescence intensity of the DCFH solution increased markedly in the presence of TPE-CSO/Rh6G/SR101, TPE-CSO/Rh6G, TPE-CSO, Rh6G + SR101, and the blank control, corresponding to 56.9-, 32.9-, 18.2-, 5.3-, and 1.7-fold enhancements, respectively. To further identify specific ROS species, 9,10-anthracenediyl-bis(methylene)-dimalonic acid (ABDA) and *N*,*N*,*N*′,*N*′-tetramethyl-phenylenediamine (TMPD) were employed as chemical probes for singlet oxygen (^1^O_2_) and superoxide anion radicals (O_2_˙^−^), respectively. Upon light irradiation, the UV-vis absorption of ABDA in various sample solutions (TPE-CSO/Rh6G/SR101, TPE-CSO/Rh6G, TPE-CSO, and Rh6G + SR101) progressively decreased (Fig. S13, SI), confirming ^1^O_2_ formation, with TPE-CSO/Rh6G/SR101 displaying the highest efficiency (Fig. 5a). Using Rose Bengal (RB) as a standard, the ^1^O_2_ quantum yield of TPE-CSO/Rh6G/SR101 was calculated to be 38.1%, outperforming TPE-CSO/Rh6G (29.0%) and TPE-CSO (9.1%) (Fig. S14 and S15, SI). Similarly, a time-dependent increase in TMPD absorption (Fig. S16, SI) indicated O_2_˙^−^ production, again most pronounced for TPE-CSO/Rh6G/SR101 (Fig. 5b).

Electron paramagnetic resonance (EPR) spectroscopy was further employed to confirm ROS generation, using 2,2,6,6-tetramethylpiperidine (TEMP) as a spin-trapping agent for ^1^O_2_ and 5,5-dimethyl-1-pyrrole-*N*-oxide (DMPO) for O_2_˙^−^ and ˙OH. Distinct EPR signals corresponding to TEMP-^1^O_2_ in water (Fig. 5c) and DMPO-O_2_˙^−^ in methanol (Fig. 5d) were clearly observed in the TPE-CSO/Rh6G/SR101 system, confirming its robust dual-generation capability for both ^1^O_2_ and O_2_˙^−^. Since ˙OH is typically produced in aqueous media, additional EPR experiments were conducted in water using DMPO as the trapping agent (Fig. S17, SI). Although the DMPO-O_2_˙^−^ signal was relatively weak in water, no discernible ˙OH signal was detected, indicating that O_2_˙^−^ remains the dominant radical species.

The temperature dependence of ROS generation in the cascade LHS was subsequently examined. As the temperature of the TPE-CSO/Rh6G/SR101 system increased from 25 °C to 60 °C, the UV-vis absorption of ABDA progressively declined (Fig. 5e and S18, SI), suggesting a marked reduction in ^1^O_2_ formation. Quantitative evaluation using RB as a reference photosensitizer revealed a sharp drop in ^1^O_2_ quantum yield, from 38.1% at 25 °C to just 5.8% at 60 °C (Fig. S19, SI), highlighting significant thermal quenching of singlet oxygen production. A comparable trend was observed for superoxide generation: the TMPD-based assay demonstrated a pronounced decline in O_2_˙^−^ production with increasing temperature (Fig. 5f and S20, SI). Together, these findings confirm the thermosensitive nature of ROS generation in the cascade LHS.

Building on the promising ROS generation performance, the photocatalytic activity of the sequential LHS was evaluated through aerobic oxidation reactions.^76–81^ The oxidative cleavage of olefins to yield carbonyl compounds is a pivotal transformation in both the chemical and pharmaceutical sectors, yet it remains challenging under ambient conditions. To demonstrate the catalytic efficacy of TPE-CSO/Rh6G/SR101, this transformation was selected as a model reaction. Using 1,1-diphenylethylene as the substrate, photocatalytic oxidation was performed in water under air with 365–375 nm light irradiation for 12 hours. Product analysis by ^1^H NMR confirmed benzophenone as the dominant product, with an impressive isolated yield of 93% (Table 1, entry 1). In contrast, control experiments yielded significantly lower conversions: 11% without photocatalyst (entry 2), 36% with only TPE-CSO (entry 3), 43% with Rh6G + SR101 (entry 4), 20% with Rh6G alone (entry 5), and 17% with SR101 alone (entry 6), highlighting the importance of energy transfer. The single-step energy transfer system TPE-CSO/Rh6G provided a moderate improvement (64%, entry 7), yet still fell short of the cascade LHS's performance. Additionally, the reaction did not proceed in the absence of light (entry 8), verifying its photo-driven nature and the critical role of the artificial LHS in facilitating the transformation. Notably, under identical conditions, direct excitation of SR101 at its optimal absorption wavelength (580 nm) also affords a 0% yield. This observation highlights the essential role of the cascade energy-transfer process and suggests that the 17% yield obtained under 365 nm excitation is mainly attributable to the direct activation of molecular oxygen rather than to SR101 excitation itself.

**Table 1:** Photocatalytic oxidation of 1,1-diphenylethene with the cascade LHS as the photocatalyst

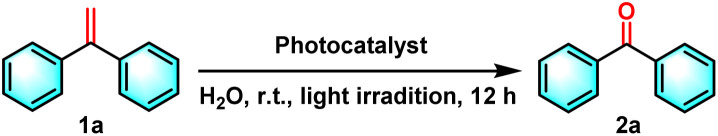		
Entry	Deviation from standard conditions	Yield^b^ (%)
1	Standard conditions^a^	93
2	No TPE-CSO/Rh6G/SR101	11
3	TPE-CSO	36
4	Rh6G + SR101	43
5	Rh6G	24
6	SR101	17
7	TPE-CSO/Rh6G	64
8	No light	NR^c^
9	30 °C instead of 25 °C	80
10	40 °C instead of 25 °C	66
11	50 °C instead of 25 °C	33
12	60 °C instead of 25 °C	27
13	TPE-CSO at 60 °C	30
14	Rh6G + SR101 at 60 °C	47

a Standard reaction conditions: 1,1-diphenylethylene (1.0 mmol), TPE-CSO/Rh6G/SR101 aqueous solution ([SR101]: 0.2 mol%, 20 mL), LED lamp (365–375 nm), room temperature, air, 12 h.

b Isolated yields.

c No reaction.

Given the thermoresponsive nature of the LHS, the impact of temperature on photocatalytic activity was further explored. Elevating the reaction temperature to 60 °C led to a progressive decline in product yield, reaching 27% (entries 9–12). This decline is attributed to the disruption of the supramolecular architecture when the temperature surpasses the LCST of the LHS. Above this threshold, the OEG chains in the TPE-CSO units undergo a hydrophilic-to-hydrophobic transition, leading to disassembly of the LHS and consequent breakdown of the cascade energy transfer pathway. The interruption of this process markedly diminishes ROS generation efficiency, thereby impairing photocatalytic activity. In addition, both TPE-CSO and Rh6G + SR101 show slight variations in their catalytic performance at 60 °C (entries 13 and 14). Notably, the system retained robust catalytic performance over 20 heating–cooling cycles between 25 °C and 60 °C, consistently achieving yields above 90% (Fig. S31, SI). This reversible thermal regulation resembles the adaptive behavior of plants, where elevated temperatures suppress photosynthetic activity, which is subsequently restored upon cooling.

To validate the applicability of the thermoresponsive cascade LHS photosensitizer, a range of styrene derivatives was subjected to photocatalytic oxidative cleavage under optimized reaction conditions. As summarized in Table 2, these substrates were efficiently transformed into the corresponding carbonyl compounds with moderate to high yields (Fig. S21–S30, SI). Notably, substrates bearing electron-donating substituents—such as phenyl, *tert*-butyl, methoxy, and naphthyl—afforded high conversion efficiencies, yielding **2b–2f** in 85%, 89%, 88%, 90%, and 91% yields, respectively. In contrast, styrene derivatives functionalized with electron-withdrawing groups (*e.g.*, bromine, carboxyl, and nitro) delivered slightly reduced yields, ranging from 71% to 84% (2g: 82%, 2h: 84%, 2i: 76%, and 2j: 71%). Importantly, when the reaction temperature was elevated to 60 °C, a pronounced decline in catalytic performance was observed across all substrates, with yields decreasing to approximately 20%. This observation highlights the critical influence of temperature on the efficiency of the artificial photosynthetic transformation.

**Table 2:** Substrate scope extension of photocatalytic oxidative reactions of alkene derivatives^a^^,^^b^

Entry	Substrate	Product	Yields	
			60 °C	25 °C
2a	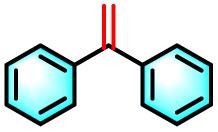	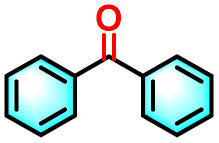	27%	93%
2b	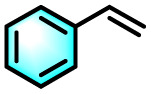	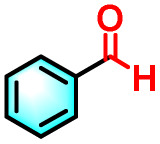	19%	85%
2c	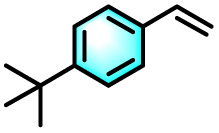	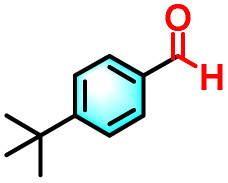	24%	89%
2d	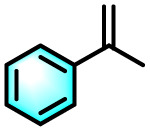	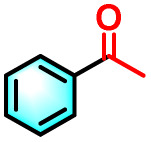	23%	88%
2e	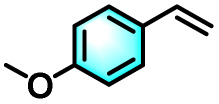	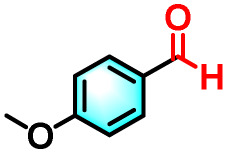	24%	90%
2f	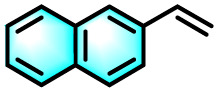	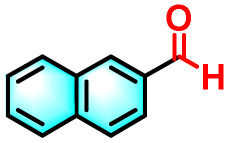	22%	91%
2g	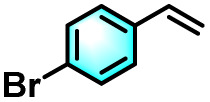	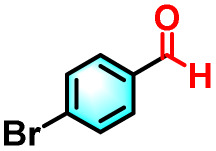	16%	82%
2h	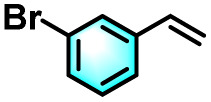	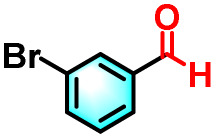	15%	84%
2i	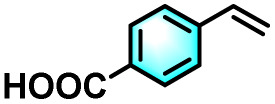	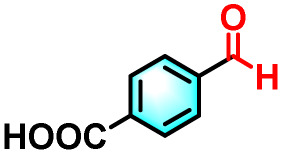	13%	76%
2j	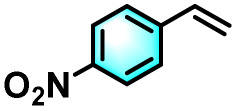	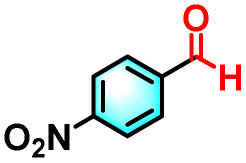	12%	71%

a Reaction conditions: styrene derivatives (1.0 mmol), TPE-CSO/Rh6G/SR101 aqueous solution ([SR101]: 0.2 mol%, 20 mL), LED lamp (365–375 nm), air, 12 h.

b Isolated yields.

To elucidate the underlying reaction mechanism and identify the predominant ROS involved in the photocatalytic oxidative cleavage of olefins into carbonyl compounds, a series of quenching experiments were systematically carried out. Selective ROS scavengers were individually introduced into the reaction medium, including potassium iodide (KI), isopropanol (IPA), 1,4-diazabicyclo[2.2.2]octane (DABCO), and 1,4-benzoquinone (*p*-BQ), which are known to quench photogenerated holes (h^+^), hydroxyl radicals (˙OH), ^1^O_2_, and O_2_˙^−^, respectively. As shown in Fig. S32 (SI), the addition of KI and IPA exerted minimal influence on product yield, which remained high (88% and 83%, respectively). These results imply that h^+^ and ˙OH play only a limited role in the catalytic process. In contrast, the presence of DABCO and *p*-BQ led to a substantial decline in yield (to 10% and 18%, respectively), indicating that ^1^O_2_ and O_2_˙^−^ are the dominant ROS facilitating the photocatalytic transformation. Drawing on these findings, a dual-pathway mechanism involving both energy transfer and electron transfer is proposed (Fig. S33, SI). Upon photoexcitation, the photosensitizer (LHS) absorbs light and transitions to its excited state (LHS*). In the energy transfer pathway, the LHS* transfers its energy to molecular oxygen (^3^O_2_), generating reactive singlet oxygen to ^1^O_2_. This ^1^O_2_ species subsequently reacts with 1,1-diphenylethylene (1a), yielding an epoxide or dioxetane intermediate (I), which decomposes to afford the final product, 1,1-diphenyl ketone (2a). Concurrently, in the electron transfer pathway, the excited photosensitizer ([LHS]*) oxidizes substrate I to produce a radical cation species (Ph_2_C=CH_2_^+^˙, II), while being reduced itself to ([LHS]˙^−^). This reduced form then transfers an electron to ^3^O_2_, generating O_2_˙^−^ and regenerating the ground-state photosensitizer. The O_2_˙^−^ subsequently reacts with intermediate II, forming the same intermediate (I), which undergoes fragmentation to yield 2a. Together, these pathways account for the observed involvement of both ^1^O_2_ and O_2_˙^−^ in the oxidative cleavage process.

## Conclusion

In summary, we have successfully developed a multifunctional artificial light-harvesting system that emulates several key features of natural photosynthesis, including sequential energy transfer, photocatalytic activity, and thermal responsiveness, all of which are intricately linked. Through the rational design and synthesis of an amphiphilic, AIE-active chromophore, TPE-CSO, we achieved supramolecular nanoparticles that function effectively as light-harvesting antennas and photosensitizers. Co-assembly with energy-matched fluorescent dyes (Rh6G and SR101) enabled the construction of a sequential energy transfer cascade, facilitating dual ROS generation (^1^O_2_ and O_2_˙^−^) and enabling the photocatalytic oxidative cleavage of 1,1-diphenylethylene derivatives with high yields. Notably, the incorporation of OEG chains endowed the system with reversible thermal sensitivity, allowing precise regulation of fluorescence intensity, ROS production, and photocatalytic activity in response to temperature fluctuations. Furthermore, the system retained high catalytic efficiency over 20 thermal cycles, demonstrating excellent robustness and reversibility, thereby mimicking the thermal deactivation behavior observed in natural photosynthetic systems. These findings highlight the potential of supramolecular design strategies for the development of adaptive, bioinspired materials and pave the way for next-generation artificial photosynthetic systems capable of intelligent environmental responses.

## Author contributions

Z. W. constructed and characterized the molecular assemblies, constructed the LHSs, synthesized all the substrates and performed the photophysical measurements. M. H. measured the ROS and investigated the reaction mechanism. G. S. revised the manuscript. T. X. designed the work, provided intellectual input, and wrote and revised the manuscript.

## Conflicts of interest

There are no conflicts to declare.

## Supplementary Material

SC-017-D5SC07946B-s001

## Data Availability

All the data supporting this article have been included in the main text and the supplementary information (SI). Supplementary information: synthetic details, additional NMR spectra, and HR-ESI-MS spectra of individual compounds. See DOI: https://doi.org/10.1039/d5sc07946b.
