# Supplementary material for: Thermoresponsive artificial light-harvesting system with temperature-gated cascade energy transfer and photocatalysis
Source: Chem Sci. 2026 Feb 17;17(15):7649–58. doi: 10.1039/d5sc07946b (PMC12931637; doi:10.1039/d5sc07946b)
Supplement: SC-017-D5SC07946B-s001 [file SC-017-D5SC07946B-s001.pdf]

Supplementary Information (SI) for Chemical Science.
This journal is © The Royal Society of Chemistry 2026

Supporting Information

Thermoresponsive artificial light-harvesting system with temperature-gated cascade energy transfer and photocatalysis

Zhiying Wu,[a] Menglian Hu,[b] Guangping Sun,[b] and Tangxin Xiao[a]*

[a] School of Petrochemical Engineering, Changzhou University, Changzhou 213164, China.

[b] School of Chemistry and Chemical Engineering, Nantong University, Nantong 226019, China.

E-mail: xiaotangxin@cczu.edu.cn

Table of Contents

1. Materials, methods, and abbreviations S2

2. Synthesis of TPE-CSO S3

3. Concentration-dependent LCST behavior S6

4. Size and morphology measurements S7

5. Temperature-dependent fluorescence emission S8

6. Construction of the two-step light-harvesting system S9

7. Temperature-dependent ROS generation S13

8. Temperature-controlled switching of photocatalysis S21

9. References S27

1. Materials, methods, and abbreviations

General. All chemicals, reagents and solvents were purchased from commercial suppliers and used, unless otherwise stated, without further purification. If needed, solvents were dried according to literature procedures. All yields were given as isolated yields. Compound **3d**[S1] and **4d**[S2] were synthesized according to literature procedure.

NMR spectroscopy. The ^{1}H NMR and ^{13}C NMR spectra were recorded in a Bruker AVANCE III (300 MHz) spectrometer and calibrated against the residual proton signal or natural abundance carbon resonance of the used deuterated solvent from tetramethylsilane (TMS) as the internal standard. The chemical shifts δ are indicated in ppm and the coupling constants J in Hz. The multiplicities are given as s (singlet), d (doublet), t (triplet) and m (multiplet).

Mass spectrometry. High-resolution electrospray ionization mass spectra (HR-ESI-MS) were recorded on an Agilent Technologies 6540 UHD Accurate-Mass.

Transmission electron microscope (TEM). TEM investigations were carried out on a JEM-2100 instrument.

Scanning electron microscope (SEM). SEM were carried out on a ZEISS SIGMA 360 instrument.

Dynamic light scattering (DLS). DLS measurements were carried out on a Brookhaven BI-9000AT system, equipped with a 200 mW polarized laser source (λ = 514 nm) at a scattering angle of 90°. All samples were prepared according to the corresponding procedures mentioned above.

UV-vis spectroscopy. UV-visible absorption spectra were measured on Shimadzu UV-1900i UV-visible spectrometer.

Fluorescence spectroscopy. Fluorescence measurements were performed on an Agilent Cary Eclipse spectrofluorometer G9800A.

Fluorescence lifetimes. The fluorescence lifetimes were measured employing time correlated single photon counting on a FLS980 instrument with a pulsed xenon lamp. Analysis of fluorescence decay curves were subjected to fit a bi-exponential decay. The instrument response function (IRF) measures the scattering of laser excitation from non-fluorescent control samples to determine the fastest possible response of the detectors.

Electron paramagnetic resonance (EPR). EPR measurements were performed using a Bruker ESR5000 spectrometer, the illumination system employed a mercury-xenon lamp (LIGHTNINGCURE LC8, Hamamatsu Photonics, Japan).

Abbreviations. CAC = critical aggregation concentration; NPs = nanoparticles; DCM = dichloromethane; MeOH = methanol; DMSO = dimethyl sulfoxide; ABDA = 9,10-anthracenediyl-bis(methylene)-dimalonic acid; TMPD = N,N,N′,N′-tetramethyl-phenylenediamine; TEMP = 2,2,6,6-tetramethylpiperidine; DMPO = 5,5-Dimethyl-1-pyrroline N-oxide; RB = Rose Bengal; *p*-BQ = 1,4-benzoquinone; DABCO = 1,4-diazabicyclo[2.2.2]octane; M = mol/L.

2. Synthesis of TPE-CSO

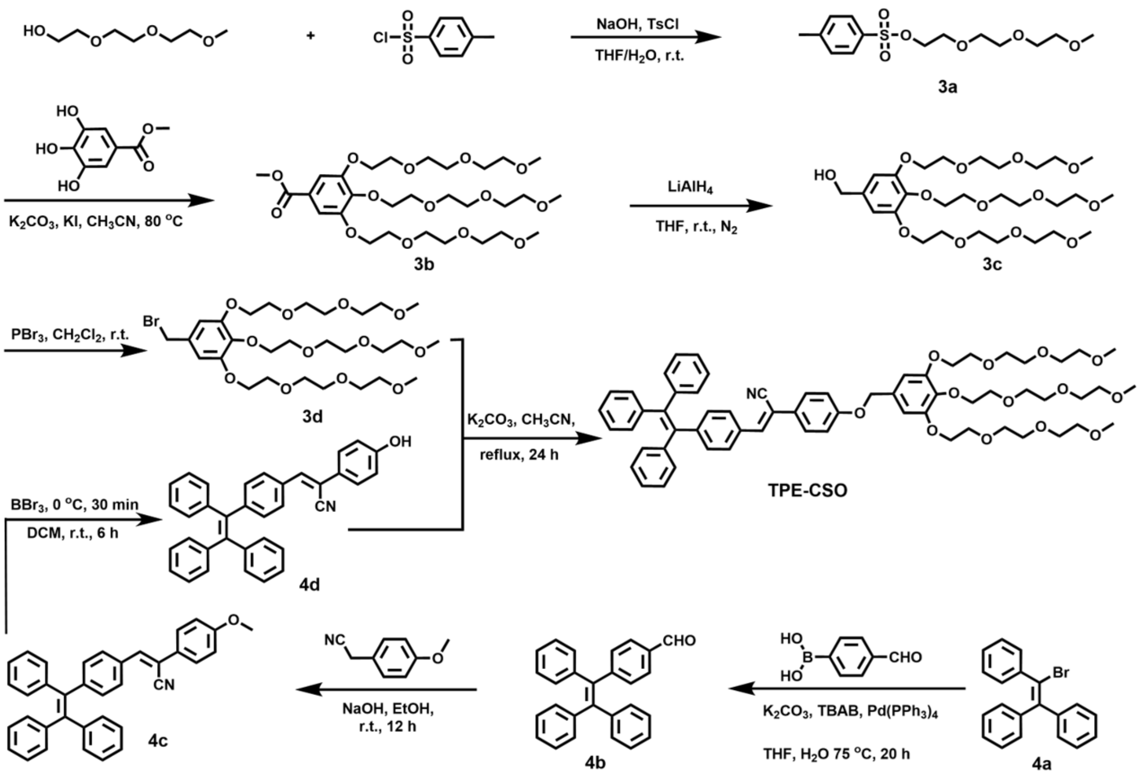

Scheme S1. Synthetic route of TPE-CSO.

Compound **3d**[S1] and **4d**[S2] were synthesized according to literature procedure.

To a flask equipped with a magnetic stirrer, K_2CO_3 (0.35 g, 2.6 mmol), compound **2d** (0.80 g, 1.7 mmol) and acetonitrile (18 mL) were charged under N_2 atmosphere. Subsequently, to the flask an acetonitrile (6 mL) solution of compound **1d** (1.23 g, 1.9 mmol) was dropped slowly. The obtained mixture was refluxed for 36 h. Then the reaction mixture was cooled down to room temperature and was concentrated under reduced pressure. The crude product was purified by silica gel column chromatography (DCM : MeOH = 200 : 1, v/v) to afford compound TPE-CSO as a viscous oil (1.00 g, 0.95 mmol, 56%). ^{1}H NMR (300 MHz, $CDCl_3$) δ (ppm) = 7.63 (d, J = 6.0 Hz, 2H, Ar-H), 7.56 (d, J = 6.0 Hz, 2H, Ar-H), 7.30 (s, 1H, -CH=C-), 7.13-7.09 (m, 11H, Ar-H), 7.07-6.98 (m, 8H, Ar-H), 6.66 (s, 2H, Ar-H), 4.98 (s, 2H, -CH_2-), 4.17-4.16 (m, 6H, -CH_2-), 3.85 (s, 4H, -CH_2-), 3.79 (s, 2H, -CH_2-), 3.73-3.72 (m, 6H, -CH_2-),

3.67-3.63 (m, 12H, -CH_2-), 3.55-3.53 (m, 6H, -CH_2-), 3.38-3.37 (m, 9H, -CH_3). ^{13}C NMR (75 MHz, $CDCl_3$): δ (ppm) =159.5, 153.0, 146.2, 143.5, 143.4, 143.3, 142.3, 140.2, 140.1, 138.4, 132.0, 131.5, 131.4, 128.6, 128.0, 127.9, 127.8, 127.6, 127.4, 127.0, 126.8, 118.4, 115.4, 110.2, 107.3, 72.4, 72.0, 70.9, 70.8, 70.7, 70.3, 69.8, 69.0, 59.1. HR-ESI-MS: *m/z* calcd for $C_{63}H_{73}NO_{13}$. $[M + Na]^+$ =1074.4974 , found =1074.4964.

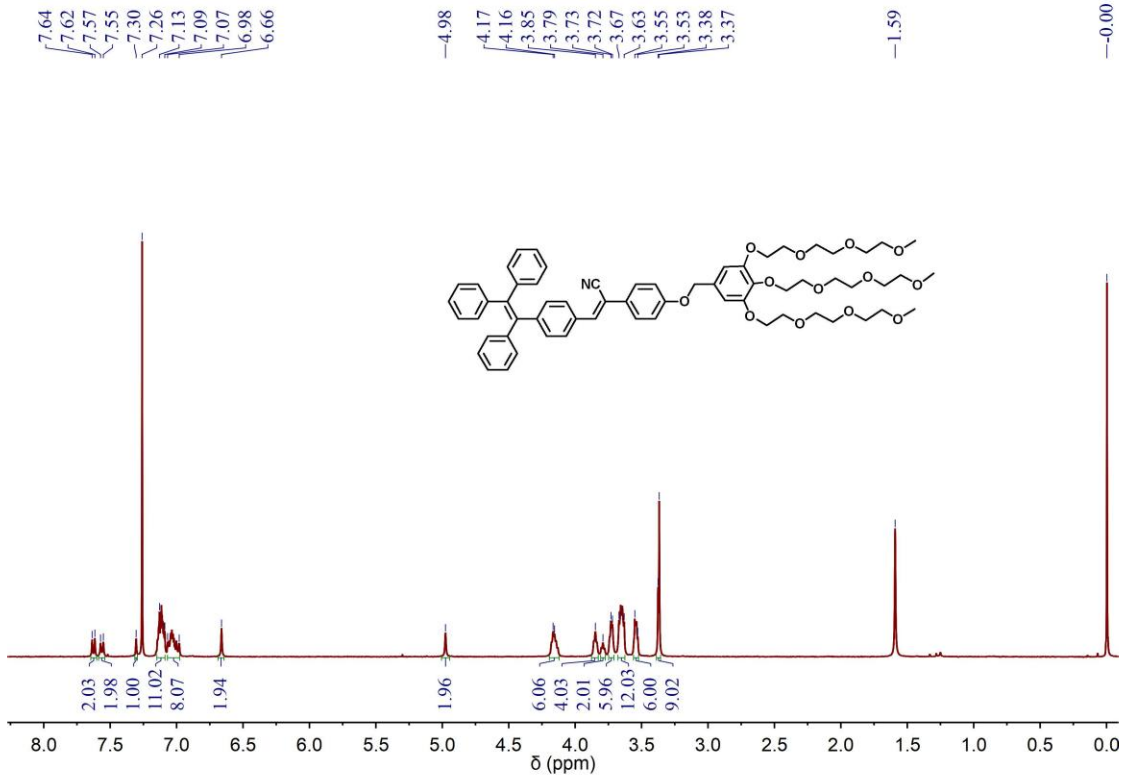

Fig. S1 ^{1}H NMR spectrum (300 MHz, $CDCl_3$, 298 K) of compound TPE-CSO.

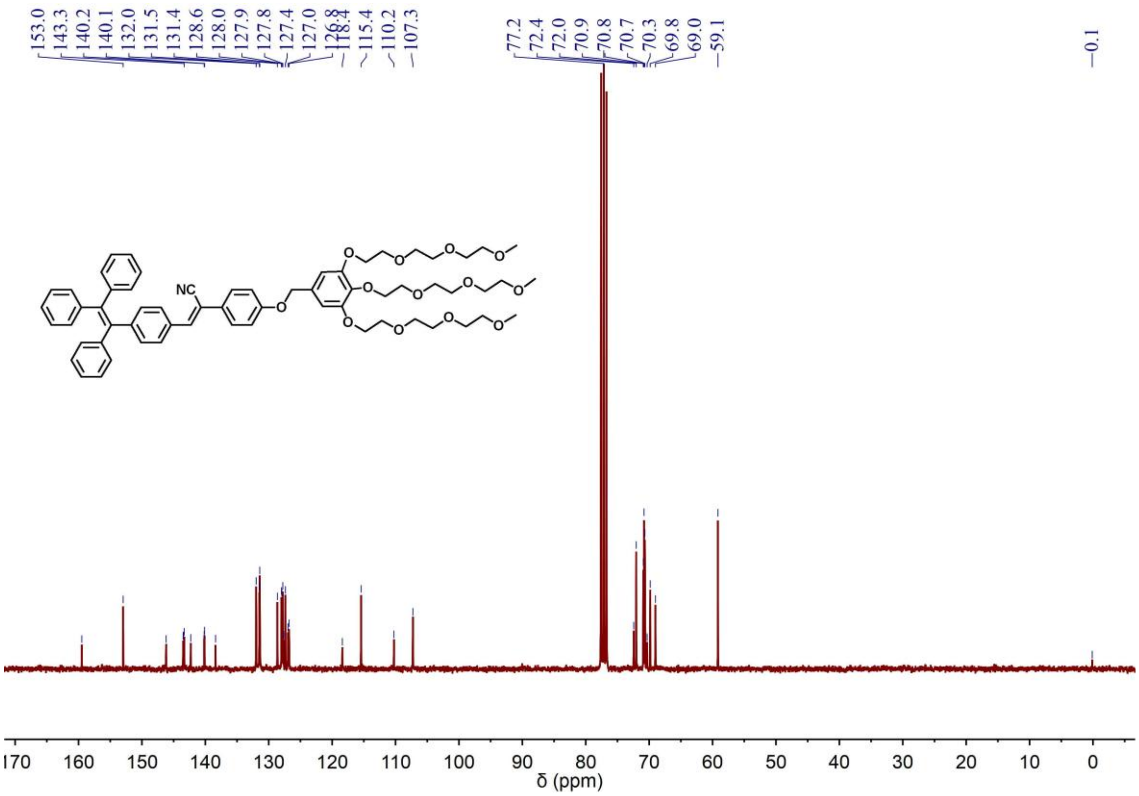

Fig. S2 ^{13}C NMR spectrum (75 MHz, $CDCl_3$, 298 K) of compound TPE-CSO.

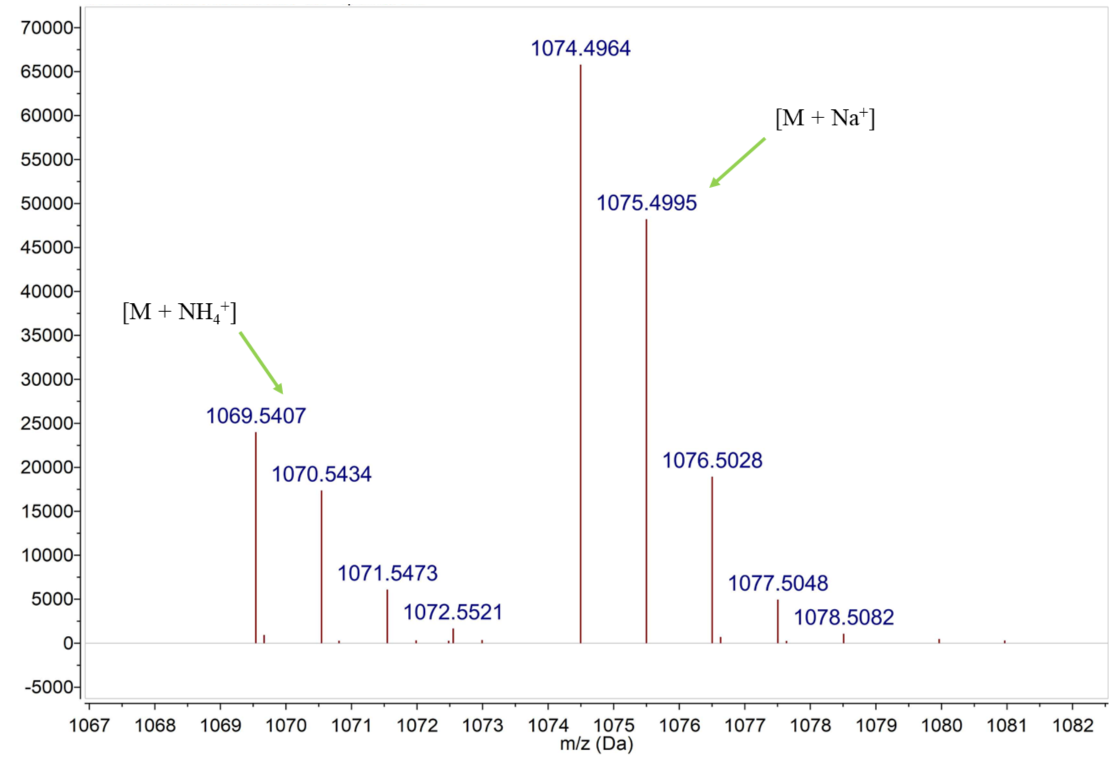

Fig. S3 HR-MS (ESI, positive mode, CH_3OH) of TPE-CSO.

3. Optical transmittance measurements

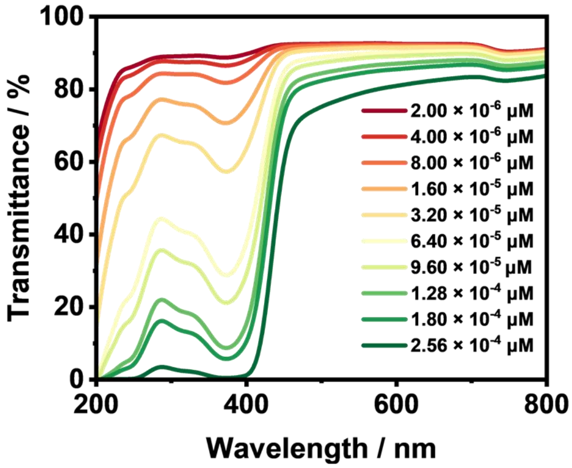

Fig. S4 Full-wavelength transmittance spectra of TPE-CSO at different concentrations.

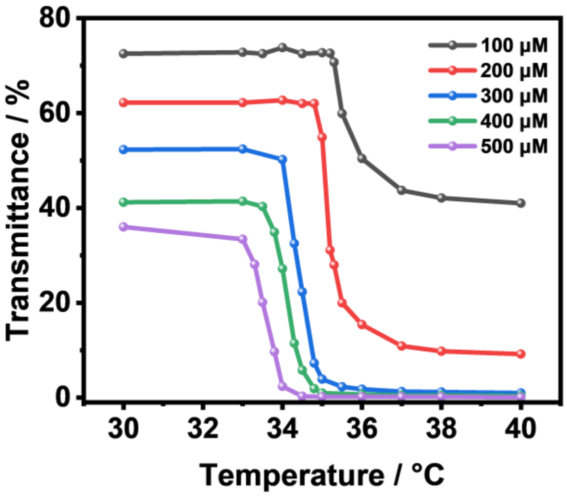

Fig. S5 Temperature-dependent optical transmittance of TPE-CSO at different concentrations.

4. Size and morphology measurements

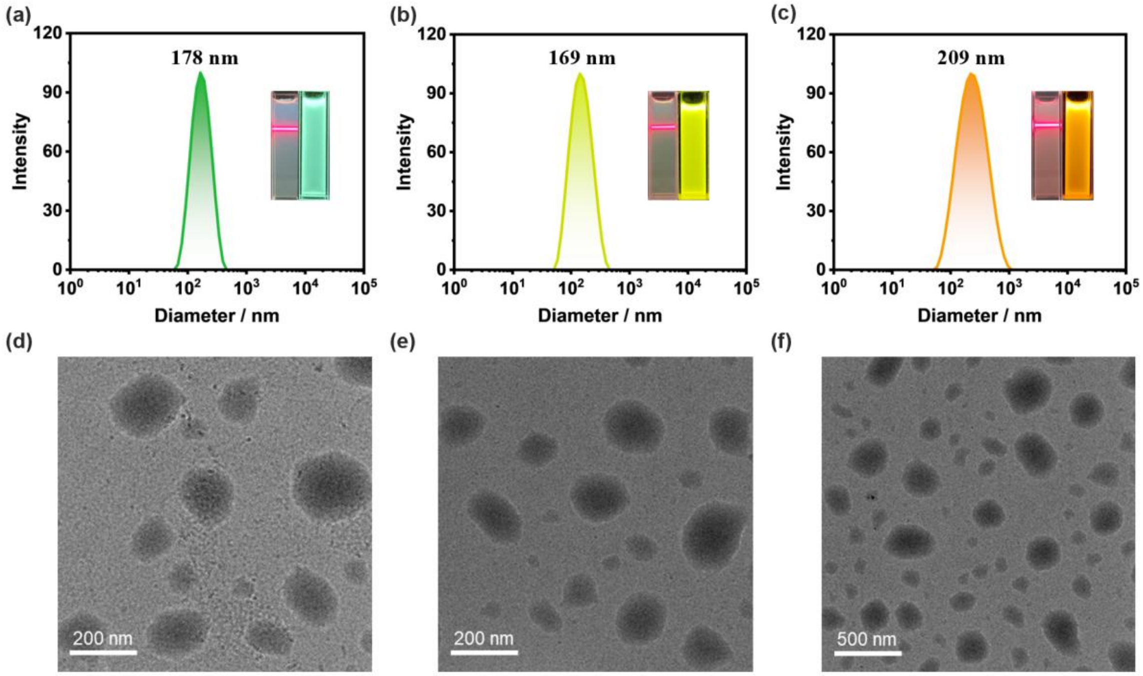

Fig. S6 DLS data of (a) TPE-CSO NPs, (b) TPE-CSO/Rh6G NPs and (c) TPE-CSO/Rh6G/SR101 NPs, insets: fluorescence picture and Tyndall effect of the corresponding samples. TEM images of (d) TPE-CSO NPs, (e) TPE-CSO/Rh6G NPs and (f) TPE-CSO/Rh6G/SR101 NPs. [TPE-CSO] = 1.0×10^{-4} M, [Rh6G] = 2.0×10^{-6} M, [SR101] = 2.0×10^{-6} M, respectively.

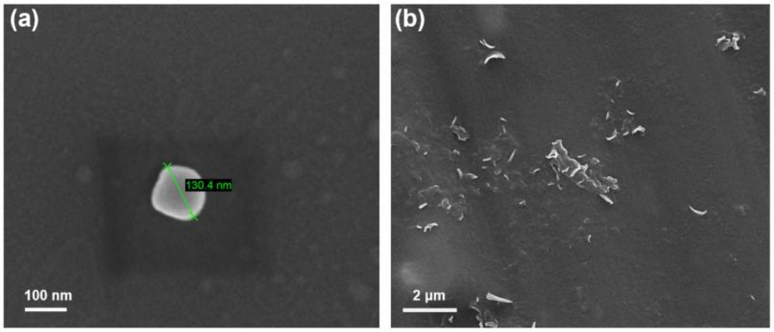

Fig. S7 SEM images of TPE-CSO obtained under (a) 25 ^{o}C and (b) 60 ^{o}C.

5. Temperature-dependent fluorescence emission

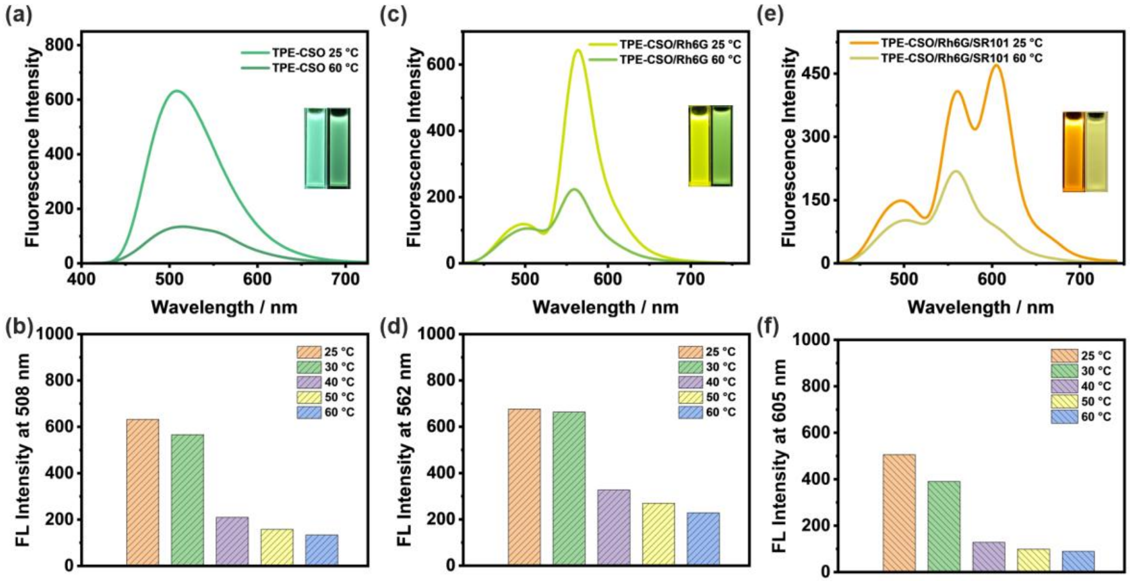

Fig. S8 Fluorescence spectra of (a) TPE-CSO, (b) TPE-CSO/Rh6G (D/A1 = 50/1), and (c) TPE-CSO/Rh6G/SR101 (D/A1/A2 = 1000/20/20) at 25 °C and 60 °C, with corresponding fluorescence images (insets). (d-f) Histograms showing temperature-dependent fluorescence intensity at 508, 562, and 605 nm, respectively.

Given the thermoresponsive nature of TPE-CSO and its influence on self-assembly, its fluorescence behavior is expected to be sensitive to temperature fluctuations due to the interplay between the AIE mechanism and its thermal responsiveness. To explore this, we investigated the temperature-dependent fluorescence characteristics of TPE-CSO. As the temperature was elevated from 25 °C to 60 °C, a gradual decrease in fluorescence intensity was observed (Fig. S8a and S8b). At ambient conditions, TPE-CSO self-assembles into well-defined, ordered nanostructures in which the TPE and CS moieties are closely packed. This rigidified arrangement restricts intramolecular motion, enhancing emission through the AIE effect. Upon heating, however, the amphiphilic balance of TPE-CSO shifts, leading to a hydrophilic-to-hydrophobic transition. This thermal response induces less ordered aggregation, facilitating increased non-radiative relaxation pathways and thereby diminishing fluorescence output. Remarkably, this fluorescence quenching is fully reversible: upon cooling back to room temperature, the emission intensity is rapidly restored, demonstrating excellent thermoresponsive reversibility. Moreover, this temperature-sensitive fluorescence behavior is retained in the co-assembled cascade LHS, in which TPE-CSO (D) is combined with Rh6G (A1) and SR101 (A2). The assembled multistep energy transfer system preserves the same reversible photophysical properties (Fig. S8c-f), further confirming the robust thermal responsiveness of the designed supramolecular LHS.

6. Construction of the two-step light-harvesting system

6.1 Fluorescence titration

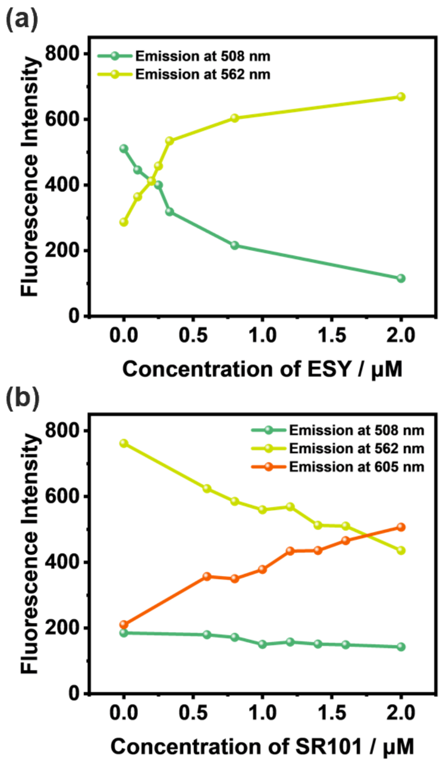

Fig. S9 Sequential energy transfer from TPE-CSO to Rh6G and SR101. (a) Fluorescence intensity changes at 508 nm and 562 nm as hydrophobic dye Rh6G is gradually loaded. (b) Fluorescence intensity changes at 508 nm, 562 nm, and 605 nm as hydrophobic dye SR101 is gradually loaded. [TPE-CSO] = 1.0×10^{-4} M, [Rh6G] = 2.0×10^{-6} M, [SR101] = 2.0×10^{-6} M.

6.2 Fluorescence lifetime

(1) The first-step FRET

Table S1 Fluorescence lifetimes of TPE-CSO and TPE-CSO/Rh6G monitored at 508 nm. [TPE-CSO] = 1.0×10^{-4} M, [Rh6G] = 2.0×10^{-6} M, respectively.

Sample	τ_1/ns	RW1[%]	τ_2/ns	RW2[%]	τ/ns	χ^2
TPE-CSO	3.43	63.16	8.35	36.84	5.25	1.0936
TPE-CSO:Rh6G = 50:1	0.89	40.12	2.74	59.88	2.00	0.9597

(2) The second-step FRET

Table S2 Fluorescence lifetimes of TPE-CSO/Rh6G and TPE-CSO/Rh6G/SR101 monitored at 562 nm. [TPE-CSO] = 1.0×10^{-4} M, [Rh6G] = 2.0×10^{-6} M, and [SR101] = 2.0×10^{-6} M, respectively.

Sample	τ_1/ns	RW1[%]	τ_2/ns	RW2[%]	τ/ns	χ^2
TPE-CSO:Rh6G = 50:1	3.55	88.82	9.68	11.18	4.24	1.1331
TPE-CSO:Rh6G:SR101 = 1000:20:20	2.26	85.08	5.10	14.92	2.68	0.8918

The energy transfer efficiency, calculated from the lifetime data using the robust formula $\Phi_{ET} = 1 - (\tau_{DA} / \tau_D)$, are Φ_{ET1} = 62% and Φ_{ET2} = 37%. These direct lifetime measurements provide definitive evidence for efficient, sequential FRET in our system. The lifetime-based efficiencies are somewhat lower than the steady-state intensity-based estimates (78% and 44%), as anticipated by the reviewer. This discrepancy is rational and commonly observed in nanostructured systems (*Nanoscale*, 2019, 11, 16284-16292; *Angew. Chem. Int. Ed.*, 2019, *58*, 1643-1647; *Angew. Chem. Int. Ed.*, 2020, *59*, 10493-10497.), as steady-state quenching can be overestimated due to inner-filter effects and static quenching contributions, whereas fluorescence lifetime is an intrinsic property largely immune to such artifacts. Utilizing the lifetime-based Φ_{ET} values, we have quantitatively determined the donor-acceptor distances for both FRET steps according to Förster theory. The average distance r is related to the efficiency Φ_{ET} and the Förster radius R_0 by $r = R_0[(1/\Phi_{ET}) - 1]^{1/6}$. The critical Förster distance R_0 was calculated from the spectral overlap integral $J(\lambda)$, the donor fluorescence quantum yield (Φ_D), the refractive index (n = 1.33), and assuming a random dipole orientation ($\kappa^2 = 2/3$). Therefore, the subsequent Förster analysis translates these efficiencies into quantitative donor-acceptor distances (~5.3 nm and ~5.5 nm), which are structurally reasonable (<10 nm) for our nanoparticle assembly and whose relationship to the calculated R_0 values perfectly corroborates the observed transfer efficiencies.

6.3 Energy-transfer efficiency

(1) The first-step FRET

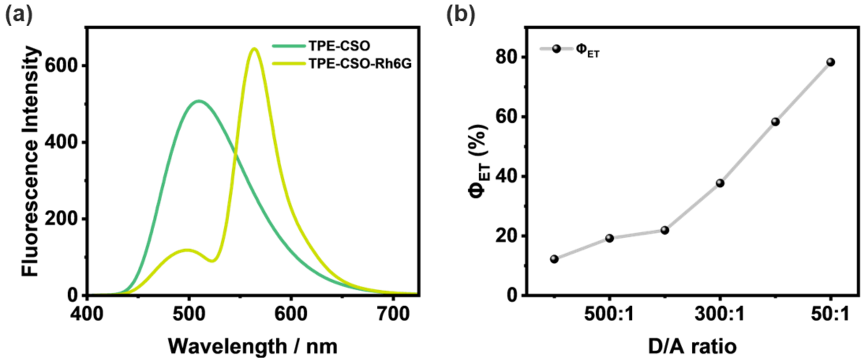

Fig. S10 Fluorescence spectra of TPE-CSO and TPE-CSO/Rh6G upon excitation at 378 nm. [TPE-CSO] = 1.0×10^{-4} M, [Rh6G] = 2.0×10^{-6} M, respectively.

Energy-transfer efficiency (Φ_{ET}) was calculated from fluorescence spectra by the equation S1[S3]:

$$\Phi_{ET} = 1 - I_{DA} / I_D \text{ (eq. S1)}$$

Where I_{DA} and I_D are the fluorescence intensities of TPE-CSO/Rh6G (donor mixed with acceptor) and TPE-CSO (individual donor) at 508 nm when excited at 378 nm, respectively.

Table S3 Energy-transfer efficiency of the one-step FRET with different TPE-CSO/Rh6G ratio.

Sample	Concentration, respectively	Energy-transfer efficiency (Φ_{ET})
TPE-CSO : Rh6G = 50 : 1	[TPE-CSO] = 1.0×10^{-4} M<br>[Rh6G] = 2.0×10^{-6} M	78.3%
TPE-CSO : Rh6G = 125 : 1	[TPE-CSO] = 1.0×10^{-4} M<br>[Rh6G] = 8.0×10^{-7} M	58.3%
TPE-CSO : Rh6G = 300 : 1	[TPE-CSO] = 1.0×10^{-4} M<br>[Rh6G] = 3.3×10^{-7} M	37.7%
TPE-CSO : Rh6G = 400 : 1	[TPE-CSO] = 1.0×10^{-4} M<br>[Rh6G] = 2.5×10^{-7} M	21.9%
TPE-CSO : Rh6G = 500 : 1	[TPE-CSO] = 1.0×10^{-4} M<br>[Rh6G] = 2.0×10^{-7} M	19.2%
TPE-CSO : Rh6G = 1000 : 1	[TPE-CSO] = 1.0×10^{-4} M<br>[Rh6G] = 1.0×10^{-7} M	12.2%

(2) The second-step FRET

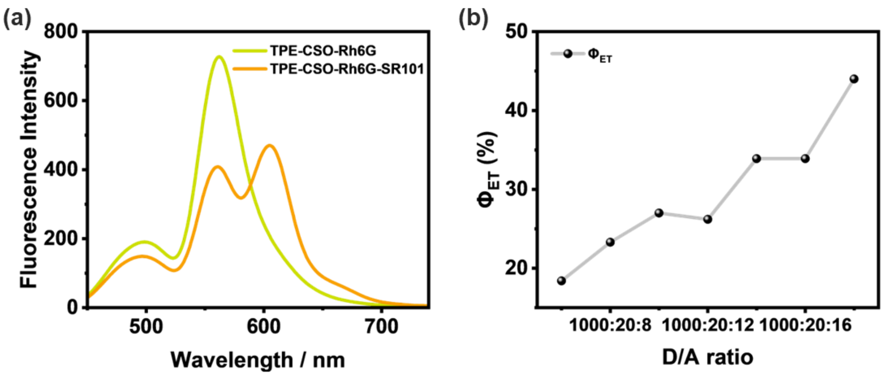

Fig. S11 Fluorescence spectra of TPE-CSO/Rh6G and TPE-CSO/Rh6G/SR101 upon excitation at 378 nm. [TPE-CSO] = 1.0 × 10^{-4} M, [Rh6G] = 2.0 × 10^{-6} M, [SR101] = 2.0 × 10^{-6} M, respectively.

Energy-transfer efficiency (Φ_{ET}) of the second-step FRET was calculated from fluorescence spectra through the equation S1[S3]:

$$\Phi_{ET} = 1 - I_{DA} / I_D \text{ (eq. S1)}$$

Where I_{DA} and I_D are the fluorescence intensities of TPE-CSO/Rh6G/SR101 and TPE-CSO/Rh6G at 562 nm when excited at 378 nm, respectively.

Table S4 Energy-transfer efficiency of the two-step FRET with different TPE-CSO/Rh6G/SR101 ratio.

Sample	Concentration, respectively	Energy-transfer efficiency (Φ_{ET})
TPE-CSO : Rh6G : SR101 = 1000 : 20 : 20	[TPE-CSO] = 1.0 × 10^{-4} M<br>[Rh6G] = 2.0 × 10^{-6} M<br>[SR101] = 2.0 × 10^{-6} M	44.0%
TPE-CSO : Rh6G : SR101 = 1000 : 20 : 16	[TPE-CSO] = 1.0 × 10^{-4} M<br>[Rh6G] = 2.0 × 10^{-6} M<br>[SR101] = 1.6 × 10^{-6} M	33.9%
TPE-CSO : Rh6G : SR101 = 1000 : 20 : 14	[TPE-CSO] = 1.0 × 10^{-4} M<br>[Rh6G] = 2.0 × 10^{-6} M<br>[SR101] = 1.4 × 10^{-6} M	33.9%
TPE-CSO : Rh6G : SR101 = 1000 : 20 : 12	[TPE-CSO] = 1.0 × 10^{-4} M<br>[Rh6G] = 2.0 × 10^{-6} M<br>[SR101] = 1.2 × 10^{-6} M	26.2%
TPE-CSO : Rh6G : SR101 = 1000 : 20 : 10	[TPE-CSO] = 1.0 × 10^{-4} M<br>[Rh6G] = 2.0 × 10^{-6} M<br>[SR101] = 1.0 × 10^{-6} M	27.0%

TPE-CSO : Rh6G : SR101 = 1000 : 20 : 8	[TPE-CSO] = 1.0 × 10^{-4} M<br>[Rh6G] = 2.0 × 10^{-7} M<br>[SR101] = 2.0 × 10^{-6} M	23.3%
TPE-CSO : Rh6G : SR101 = 1000 : 20 : 6	[TPE-CSO] = 1.0 × 10^{-4} M<br>[Rh6G] = 2.0 × 10^{-6} M<br>[SR101] = 6.0 × 10^{-7} M	18.4%

7. Temperature-dependent ROS generation

7.1 Detection of the overall ROS generation

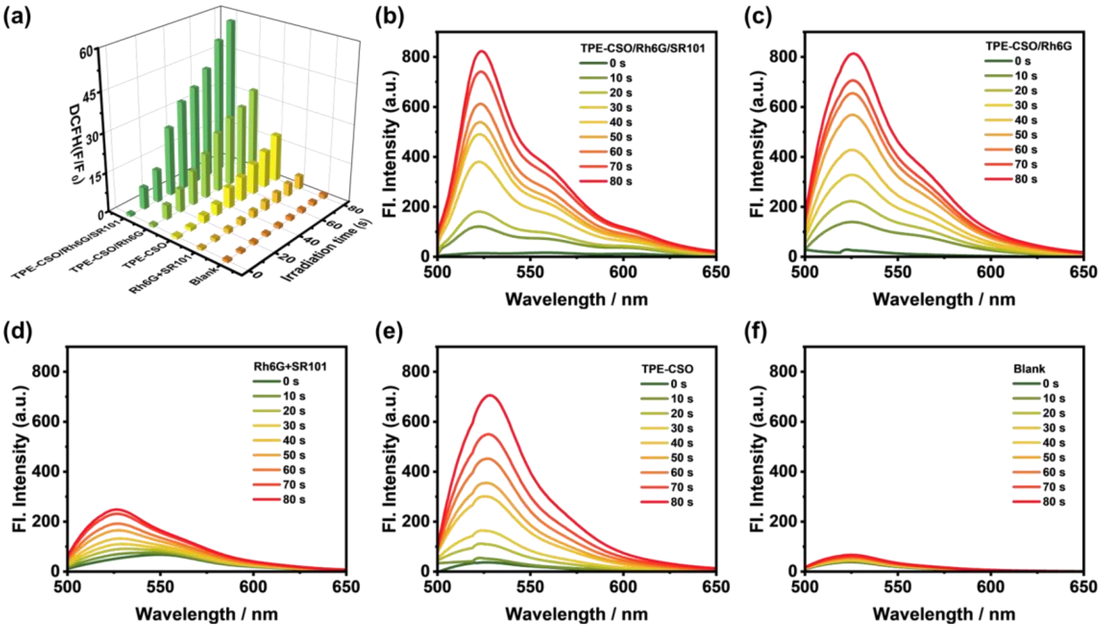

Fig. S12 Histogram of (a) F/F_0 for DCFH at 523 nm upon light irradiation for different times in the presence of TPE-CSO/Rh6G/SR101, TPE-CSO/Rh6G, Rh6G + SR101 mixture, and TPE-CSO (blank: DCFH without any additive). Fluorescence spectra of DCFH under light irradiation for different times and the experimental conditions are as follows: (b) TPE-CSO/Rh6G/SR101, (c) TPE-CSO/Rh6G, (d) Rh6G + SR101, (e) TPE-CSO, (f) blank. [DCFH] = 5.0 × 10^{-5} M, [TPE-CSO] = 5.0 × 10^{-5} M, [Rh6G] = 1.0 × 10^{-6} M, [SR101] = 1.0 × 10^{-6} M, V(H_2O) = 3 mL.

Compound 2,7-dichlorodihydrofluorescein (DCFH) was used as an indicator for the detection of the overall ROS generation in aqueous solution (Fig. S12)[S4]. 5.0 × 10^{-5} M of photosensitizer was

dissolved in 3.0 mL solution containing 5.0×10^{-5} M of DCFH. The mixture was then placed in a cuvette and irradiated with light (365-375 nm, 10 W). The change of the emission intensity of the sample at 523 nm with irradiation time was recorded by fluorescence emission spectrum. Various photosensitizer systems were tested, including TPE-CSO/Rh6G/SR101 (LHS), TPE-CSO/Rh6G, TPE-CSO, Rh6G + SR101, and the blank group. Notably, the emission peak of DCFH in the cascaded LHS strengthen most significantly, followed by TPE-CSO/Rh6G, TPE-CSO, and Rh6G + SR101. In the absence of a photosensitizer, the emission peak of DCFH showed little change with irradiation time. These results indicate that the cascaded LHS has the strongest ability to generate ROS in a short time (80 s).

7.2 Detection of $^1O_2$ production

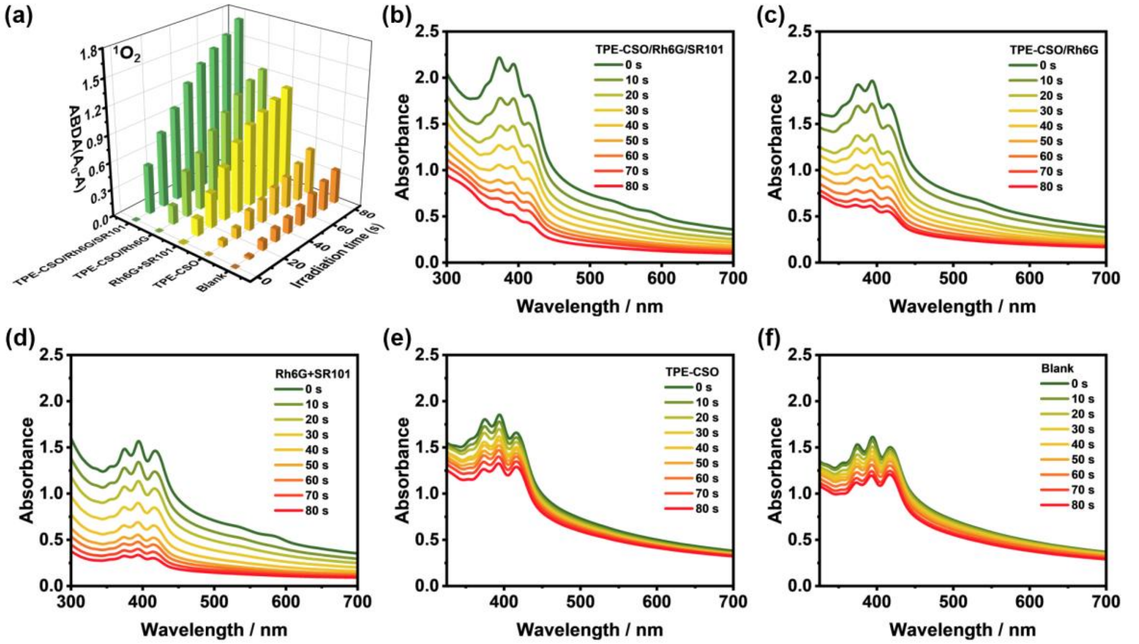

Fig. S13 Histogram of (a) ΔAbs (A_0-A) for ABDA at 371 nm upon light irradiation (365-375 nm, 10 W) for different times in the presence of TPE-CSO/Rh6G/SR101, TPE-CSO/Rh6G, Rh6G + SR101 mixture, and TPE-CSO (blank: ABDA without any additive). Absorption spectra of ABDA under light irradiation for different times, and the experimental conditions are as follows: (b) TPE-CSO/Rh6G/SR101, (c) TPE-CSO/Rh6G, (d) Rh6G + SR101, (e) TPE-CSO, (f) blank. [ABDA] = 5.0×10^{-4} M, [TPE-CSO] = 5.0×10^{-5} M, [Rh6G] = 1.0×10^{-6} M, [SR101] = 1.0×10^{-6} M, V(H_2O) = 3 mL.

Compound 9,10-anthracenediyl-bis(methylene)-dimalonic acid (ABDA) was used as an indicator for the detection of $^1O_2$ in aqueous solution (Fig. S13)[S5]. Photosensitizer (5.0×10^{-5} M) was dissolved in 3.0 mL solution containing 5.0×10^{-4} M of ABDA. The mixture was then placed in a cuvette and irradiated with light. The decrease of the absorption intensity of the sample at 371 nm with irradiation time was recorded by UV-vis absorption spectrophotometer. Notably, the absorption peak of ABDA in

the cascaded LHS decreased most significantly, followed by TPE-CSO/Rh6G, Rh6G/SR101, and TPE-CSO. These results indicate that the cascaded LHS as a photocatalyst has the strongest ability to generate $^1O_2$ in a short time (80 s).

7.3 Quantum yield detection of singlet oxygen

Rose Bengal (RB) was selected as a reference photosensitizer for the determination of singlet oxygen quantum yield (Fig. S14). An aqueous solution of RB (3.0 mL, 5.0×10^{-5} M) was prepared, containing 5.0×10^{-4} M ABDA. The mixed solution was then placed in a test tube and exposed to light irradiation at wavelengths of 365-375 nm (10 W). UV-vis spectrophotometer was used to monitor the absorbance at 371 nm over time. The $^1O_2$ quantum yield of the system was then calculated by comparing it with the reference system.

The quantum yield of $^1O_2$ was quantitatively calculated based on absorption intensity using equation S2[S5].

$$\Phi_{probe} = \Phi_{RB} \times (K_{probe} \times A_{RB} / K_{RB} \times A_{probe}) \quad \text{(eq. S2)}$$

Here, K_{probe} and K_{RB} represent the decomposition rate constants of ABDA in the presence of the probe (photosensitizer) and RB, respectively. Φ_{RB} denotes the quantum yield of $^1O_2$ generated by RB in aqueous solution ($\Phi_{RB} = 0.75$). A_{probe} and A_{RB} correspond to the integrated absorption peak areas of the probe and RB within the 365-375 nm wavelength range.

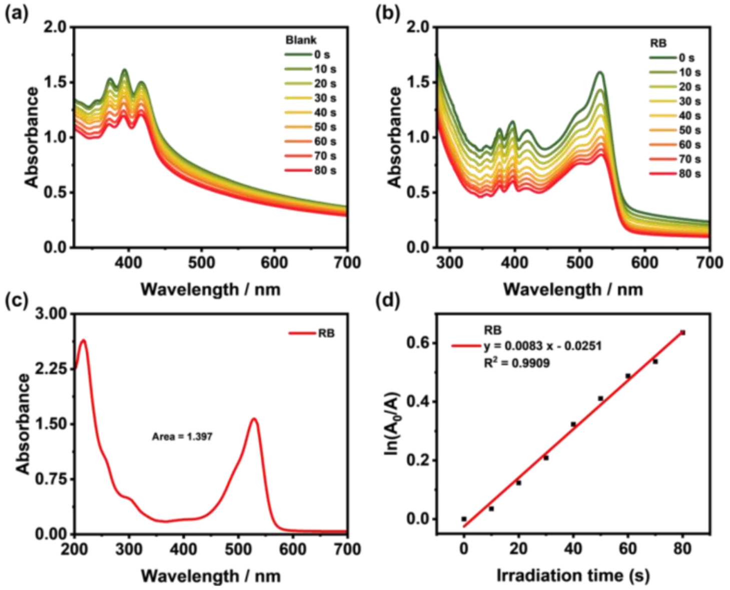

Fig. S14 (a) absorption spectra of ABDA alone after different irradiation times; (b) absorption spectra of ABDA in the presence of RB after different irradiation times; (c) absorption spectrum of RB in aqueous solution; (d) the linear relationship between the decomposition rate of ABDA and the radiation time in the presence of RB.

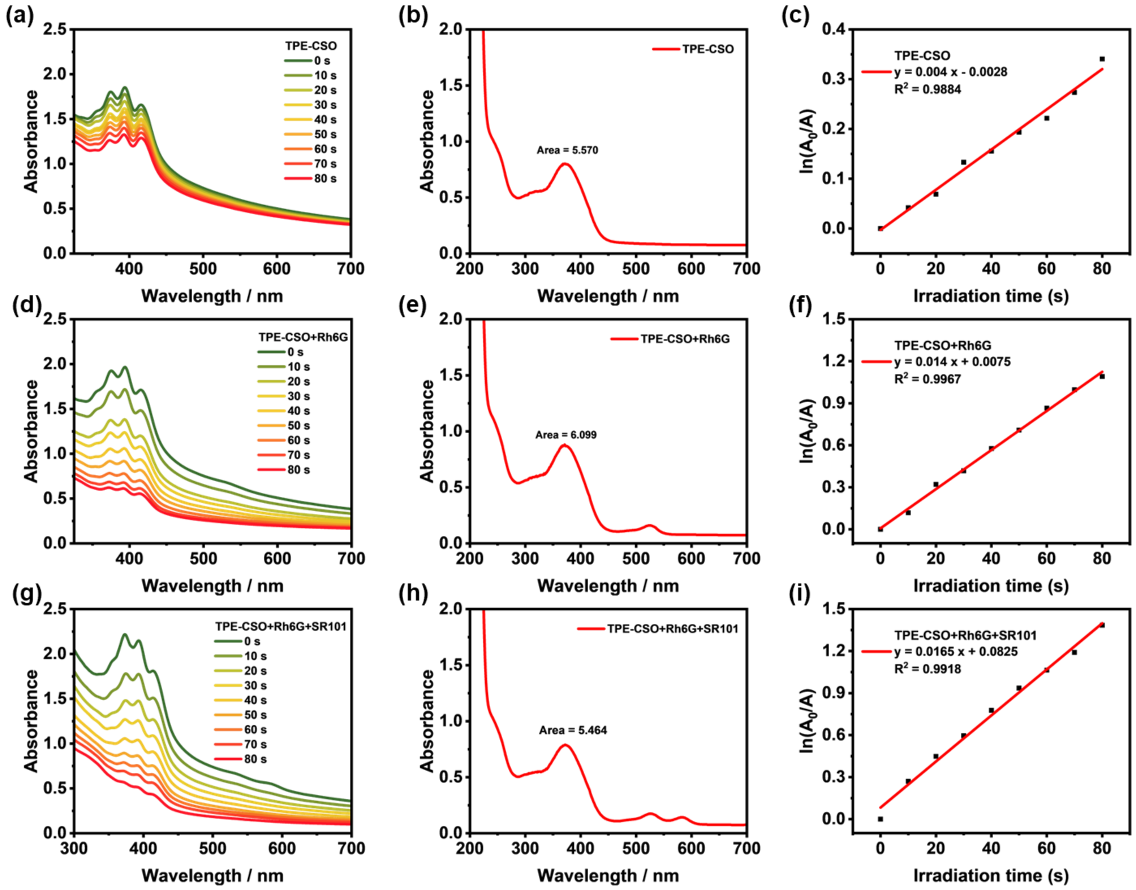

Fig. S15 Absorption spectra of ABDA (5.0×10^{-4} M) after different irradiation times in the presence of (a) TPE-CSO, (d) TPE-CSO/Rh6G, and (g) TPE-CSO/Rh6G/SR101; absorption spectra of (b) TPE-CSO, (e) TPE-CSO/Rh6G, and (h) TPE-CSO/Rh6G/SR101 in aqueous solution; Linear relationship between ABDA degradation rate and irradiation time in the presence of (c) TPE-CSO, (f) TPE-CSO/Rh6G and (i) TPE-CSO/Rh6G/SR101.

As shown in Fig. S14 and S15, the absorption peak of ABDA at 371 nm gradually decreased with increasing irradiation time. To evaluate the photosensitizing activity of different systems, various photosensitizers were tested, including TPE-CSO/Rh6G/SR101, TPE-CSO/Rh6G, TPE-CSO, the reference photosensitizer RB, and a blank control. By analyzing the absorption spectra of these systems and calculating the $^1O_2$ quantum yield using equation S2[S4], it was determined that the cascaded LHS exhibited the highest quantum yield of 38.1%, followed by TPE-CSO/Rh6G (29.0%) and TPE-CSO (9.1%). These results indicate that the cascaded LHS functions as an efficient photosensitizer, capable of producing $^1O_2$ with high efficiency within a short irradiation time (80 s).

7.4 Detection of $O_2^{\bullet-}$ production

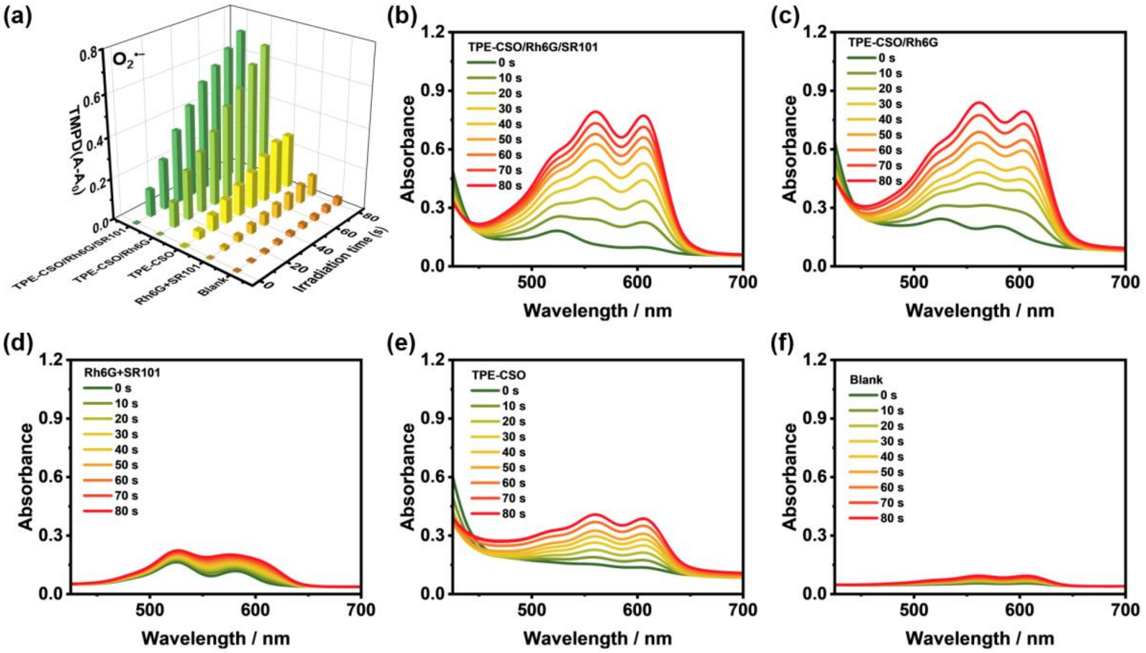

Fig. S16 Histogram of (a) ΔAbs (A-A_0) for TMPD at 560 nm upon light irradiation (365-375 nm, 10 W) for different times in the presence of TPE-CSO/Rh6G/SR101, TPE-CSO/Rh6G, Rh6G + SR101 mixture, and TPE-CSO (blank: TMPD without any additive). Absorption spectra of TMPD under light irradiation for different times, with experimental conditions as follows: (b) TPE-CSO/Rh6G/SR101, (c) TPE-CSO/Rh6G, (d) Rh6G + SR101, (e) TPE-CSO, (f) blank. [TMPD] = 5.0×10^{-4} M, [TPE-CSO] = 5.0×10^{-5} M, [Rh6G] = 1.0×10^{-6} M, [SR101] = 1.0×10^{-6} M, V(H_2O) = 3 mL.

N,*N*,*N*′,*N*′-tetramethyl-phenylenediamine (TMPD) was used as an indicator for detection of $O_2^{\bullet-}$ in its aqueous solution[S5]. The photosensitizer (5.0×10^{-5} M) was dissolved in 3.0 mL solution containing 5.0×10^{-4} M of TMPD. The mixture was then placed in a cuvette and irradiated with the light. The change of the absorption intensity of the sample at 560 nm with irradiation time was recorded by UV-vis absorption spectrophotometer. As shown in Fig. S16, the absorption peak of TMPD at 560 nm continuously increased with the increase in irradiation time. The photosensitizers TPE-CSO/Rh6G/SR101, TPE-CSO/Rh6G, TPE-CSO, Rh6G + SR101, and the blank were tested respectively. It is noteworthy that in the cascaded LHS system, the absorption peak of TMPD showed the most significant increase, followed by TPE-CSO/Rh6G, TPE-CSO, and Rh6G + SR101. In the absence of a photosensitizer, the change of the absorption peak of TMPD is negligible. Overall, these results indicate that the cascaded LHS as a photosensitizer has the strongest ability to generate $O_2^{\bullet-}$ in a short time (80 s).

7.5 EPR measurements

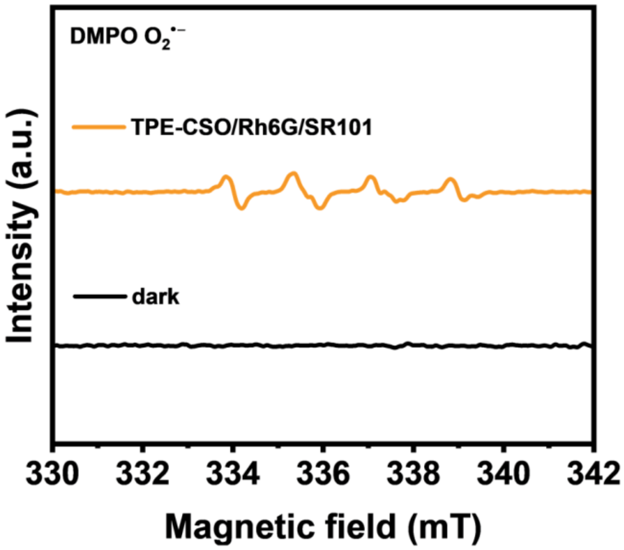

Fig. S17 EPR patterns of TPE-CSO/Rh6G/SR101 with DMPO in water under light and dark.

7.6 Effect of temperature on the production of $^1O_2$

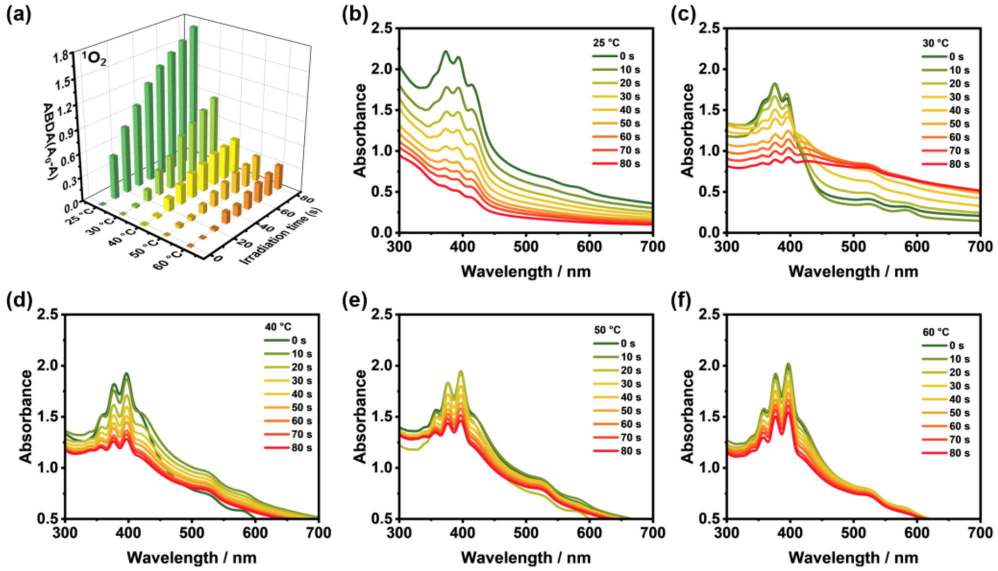

Fig. S18 Histogram of temperature-dependent (a) ΔAbs (A_0-A) for ABDA at 371 nm upon light irradiation for different times in the presence of TPE-CSO/Rh6G/SR101. Absorption spectra of ABDA under light irradiation for different times under different temperatures: (b) 25 °C, (c) 30 °C, (d) 40 °C, (e) 50 °C, (f) 60 °C. [ABDA] = 5.0 × 10^{-4} M, [TPE-CSO] = 5.0 × 10^{-5} M, [Rh6G] = 1.0 × 10^{-6} M, [SR101] = 1.0 × 10^{-6} M, V(H_2O) = 3 mL.

The mixture was placed in a cuvette and irradiated with light at 30 °C, 40 °C, 50 °C, 60 °C water bath, respectively. The change in absorption intensity at 371 nm with irradiation time at different temperature was recorded. As shown in Fig. S18, the absorption peak of ABDA at 371 nm gradually decreases as the temperature increases from 25 °C to 60 °C, indicating that the ability of the photosensitizer to generate $^1O_2$ is significantly inhibited by temperature.

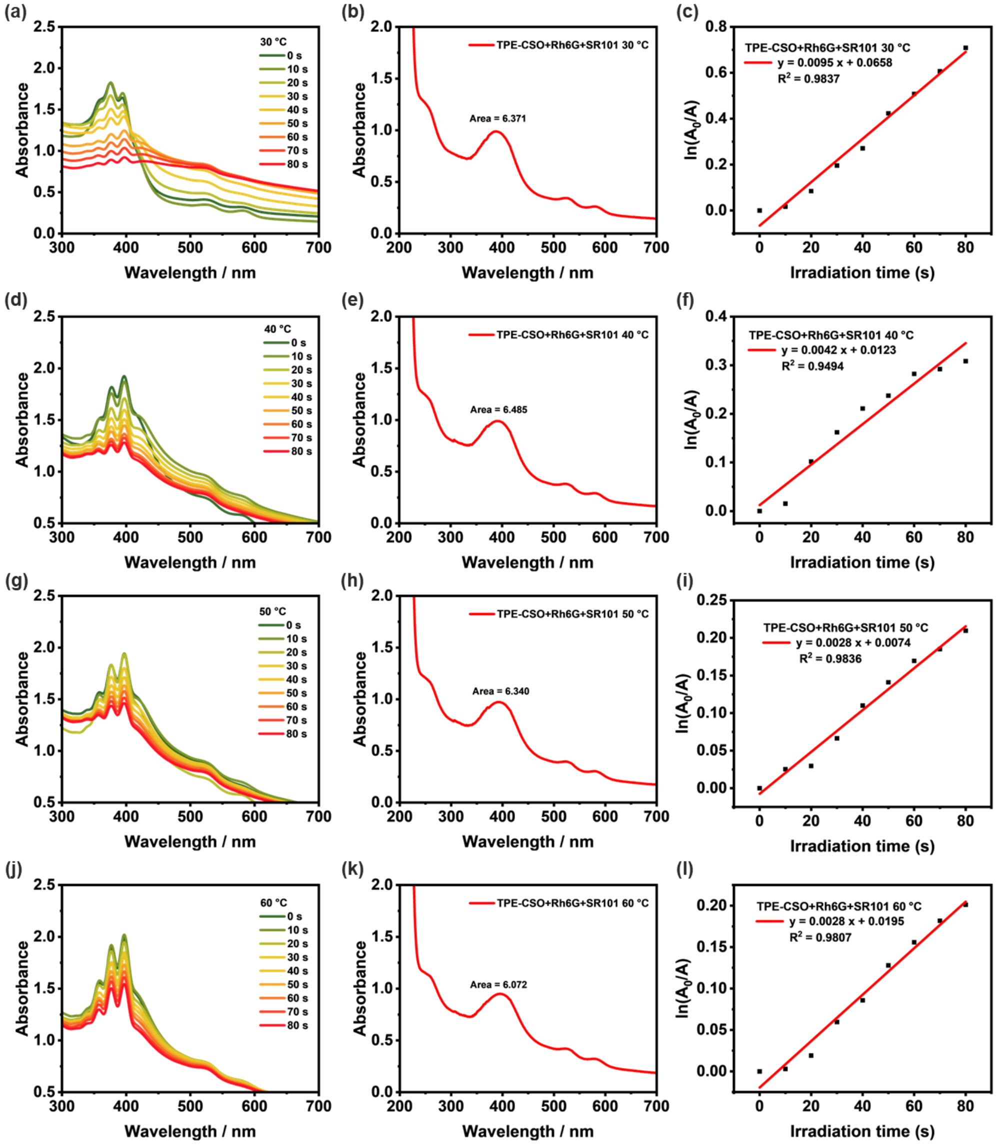

Fig. S19 Absorption spectra of ABDA (5.0×10^{-4} M) in the presence of the thermosensitive LHS under different temperature (a) 30 °C, (d) 40 °C, (g) 50 °C, and (j) 60 °C. Absorption spectra of TPE-CSO/Rh6G/SR101 (5.0×10^{-5} M) under different temperature (b) 30 °C, (e) 40 °C, (h) 50 °C, and (k) 60 °C. Linear relationship between ABDA degradation rate and irradiation time at different temperatures: (c) 30 °C, (f) 40 °C, (i) 50 °C, and (l) 60 °C.

Furthermore, the quantum yield of $^1O_2$ generation at different temperatures was investigated. According to equation S2[S5], the quantum yield of $^1O_2$ for the cascaded LHS was calculated to be 25 °C (38.1%), 30 °C (18.8%), 40 °C (8.2%), 50 °C (5.6%), and 60 °C (5.8%). These results indicate that the LHS exhibits the highest $^1O_2$ quantum yield at room temperature, while its ability to generate $^1O_2$ decreases progressively as the temperature increases.

7.7 Effect of temperature on the production of $O_2^{•-}$

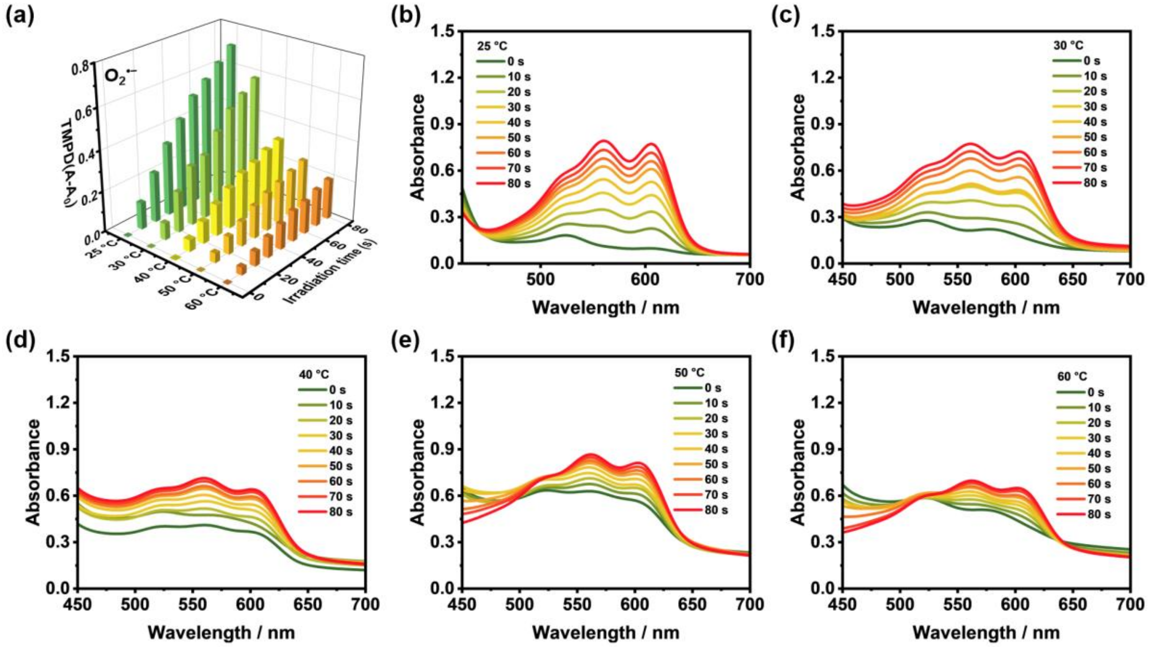

Fig. S20 Histogram of temperature-dependent (a) ΔAbs (A-A_0) for TMPD at 560 nm upon light irradiation for different times in the presence of TPE-CSO/Rh6G/SR101. Absorption spectra of TMPD under light irradiation for different times at different temperatures: (b) 25 °C, (c) 30 °C, (d) 40 °C, (e) 50 °C, (f) 60 °C. [TMPD] = 5.0×10^{-4} M, [TPE-CSO] = 5.0×10^{-5} M, [Rh6G] = 1.0×10^{-6} M, [SR101] = 1.0×10^{-6} M, V(H_2O) = 3 mL.

We further investigated the effect of temperature on $O_2^{•-}$ production. The sample was placed in a cuvette and irradiated with light under 30 °C, 40 °C, 50 °C, and 60 °C, respectively. The absorption intensity at 560 nm was recorded as a function of irradiation time in different temperature environments. As shown in Fig. S20, the absorption peak of TMPD at 560 nm decreases gradually as the temperature increases from 25 °C to 60 °C. The results indicate that the ability of the photosensitizer to produce $O_2^{•-}$ is inhibited with increasing temperature.

8. Temperature-controlled switching of photocatalysis

8.1 Photocatalytic oxidation procedure

1,1-Diphenylethylene (180 μL, 1.0 mmol) was dispersed in freshly prepared photocatalyst (TPE-CSO/Rh6G/SR101, for example, [TPE-CSO] = 1.0×10^{-4} M, [Rh6G] = 2.0×10^{-6} M, [SR101] = 2.0×10^{-6} M) aqueous solution (20 mL). The mixture was then irradiated with light (365-375 nm) for 12 h at room temperature. After irradiation, the organic phase was extracted with ethyl acetate, and the combined organic layer was dried with anhydrous Na_2SO_4. The crude product was concentrated under reduced pressure and purified by column chromatography (PE/DCM = 5/1) to obtain a white solid (0.17 g, 0.93 mmol, 93%), namely 1,1-benzophenone. The corresponding derivatives were also synthesized using the same reaction method.

8.2 ^{1}H NMR data of 2a-2j

2a: benzophenone, white solid, 93% yield, ^{1}H NMR (300 MHz, $CDCl_3$, 298 K) : *δ* (ppm) = 7.82 ~ 7.79 (m, 4H, Ar-*H*), 7.62 ~ 7.57 (m, 2H, Ar-*H*), 7.51 ~ 7.46 (m, 4H, Ar-*H*).

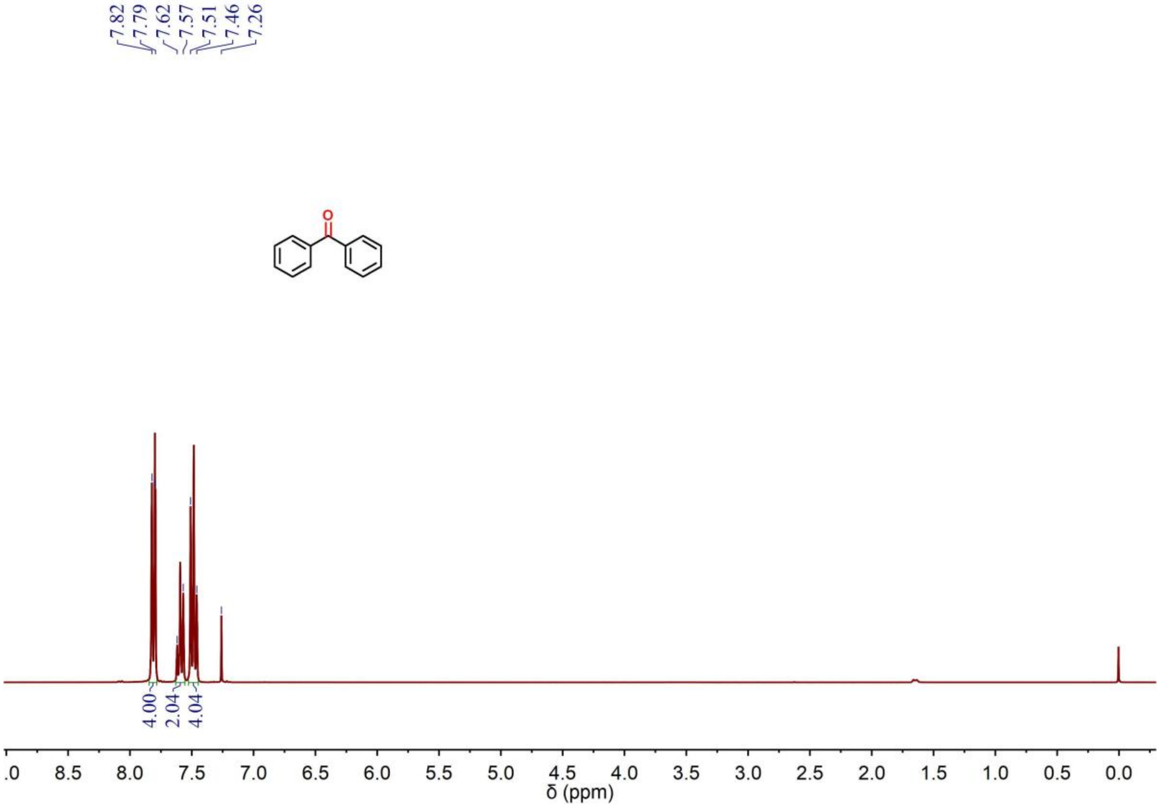

Fig. S21 ^{1}H NMR spectrum (300 MHz, $CDCl_3$, 298 K) of **2a**.

2b: benzaldehyde, colorless oil liquid, 85% yield, ^{1}H NMR (300 MHz, $CDCl_3$, 298 K) : δ (ppm) = 10.02 (s, 1H, -C*H*O), 7.89 ~ 7.86 (m, 2H, Ar-*H*), 7.66 ~ 7.60 (m, 1H, Ar-*H*), 7.55 ~ 7.50 (m, 2H, Ar-*H*).

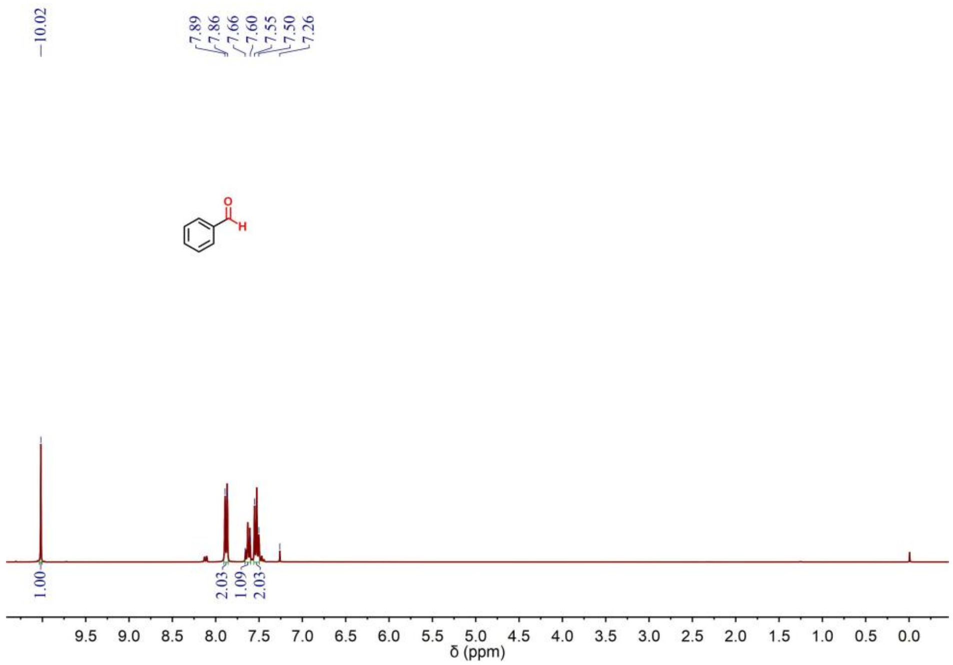

Fig. S22 ^{1}H NMR spectrum (300 MHz, $CDCl_3$, 298 K) of **2b**.

2c: 4-tert-butylbenzaldehyde, pale yellow oily liquid, 89% yield, ^{1}H NMR (300 MHz, $CDCl_3$, 298 K) : δ (ppm) = 9.98 (s, 1H, -C*H*O), 7.82 (d, J = 9.0 Hz, 2H, Ar-*H*), 7.55 (d, J = 6.0 Hz, 2H, Ar-*H*), 1.35 (s, 9H, -CH_3).

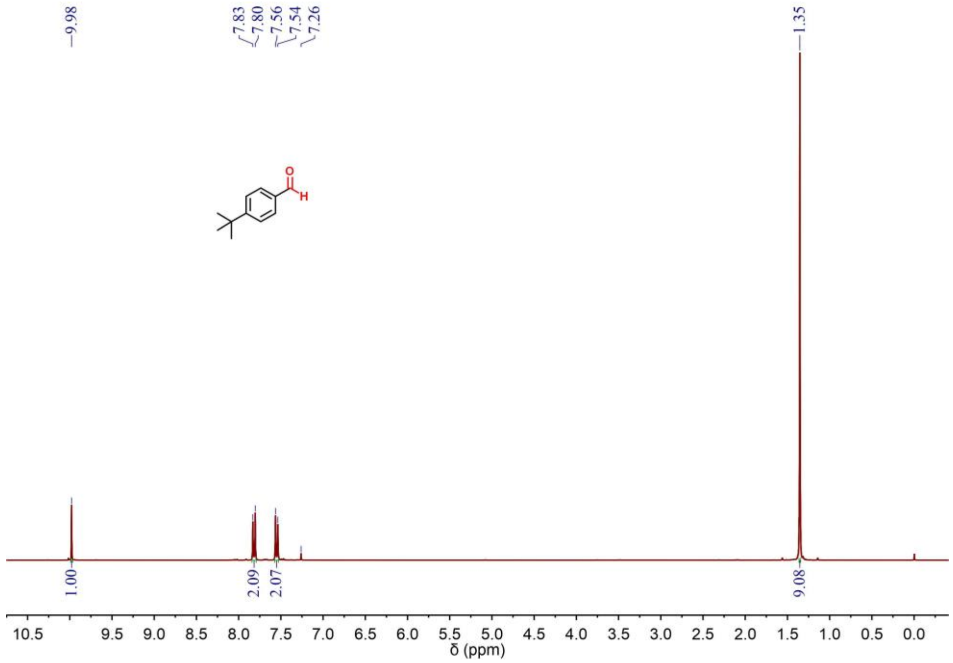

Fig. S23 ^{1}H NMR spectrum (300 MHz, $CDCl_3$, 298 K) of **2c**.

2d: acetophenone, colorless oil liquid, 88% yield, ^{1}H NMR (300 MHz, $CDCl_3$, 298 K) : δ (ppm) = 7.95 ~ 7.93 (m, 2H, Ar-*H*), 7.54 ~ 7.44 (m, 3H, Ar-*H*), 2.59 (s, 3H, -CH_3).

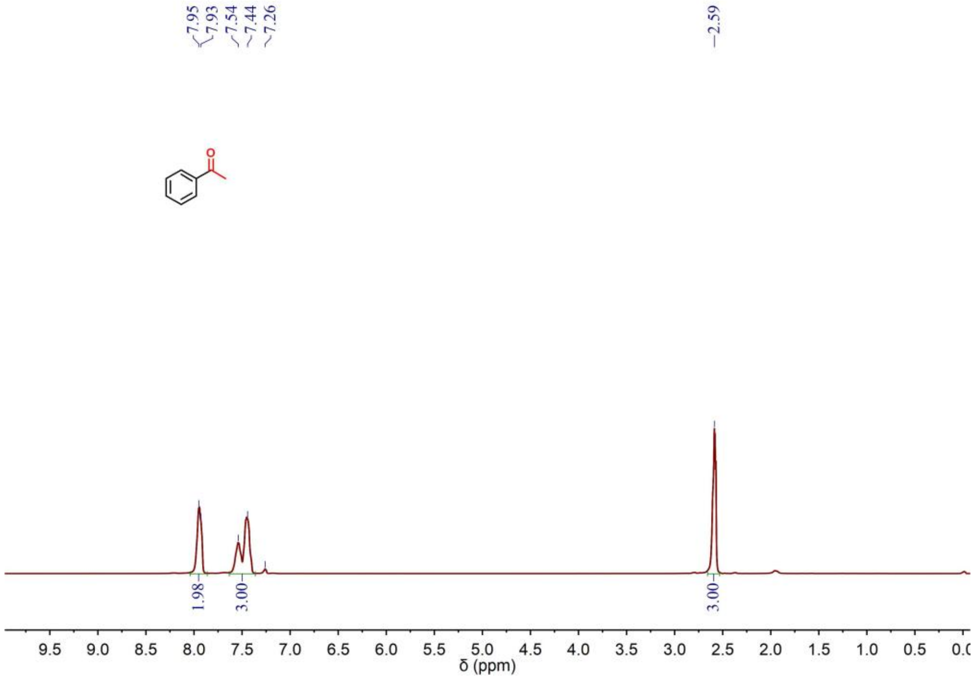

Fig. S24 ^{1}H NMR spectrum (300 MHz, $CDCl_3$, 298 K) of **2d**.

2e: 4-methoxybenzaldehyde, colorless oil liquid, 90% yield, ^{1}H NMR (300 MHz, $CDCl_3$, 298 K) : δ (ppm) = 9.86 (s, 1H, -C*H*O), 7.82 (d, *J* = 9.0 Hz, 2H, Ar-*H*), 6.99 (d, *J* = 9.0 Hz, 2H, Ar-*H*), 3.86 (s, 3H, -CH_3).

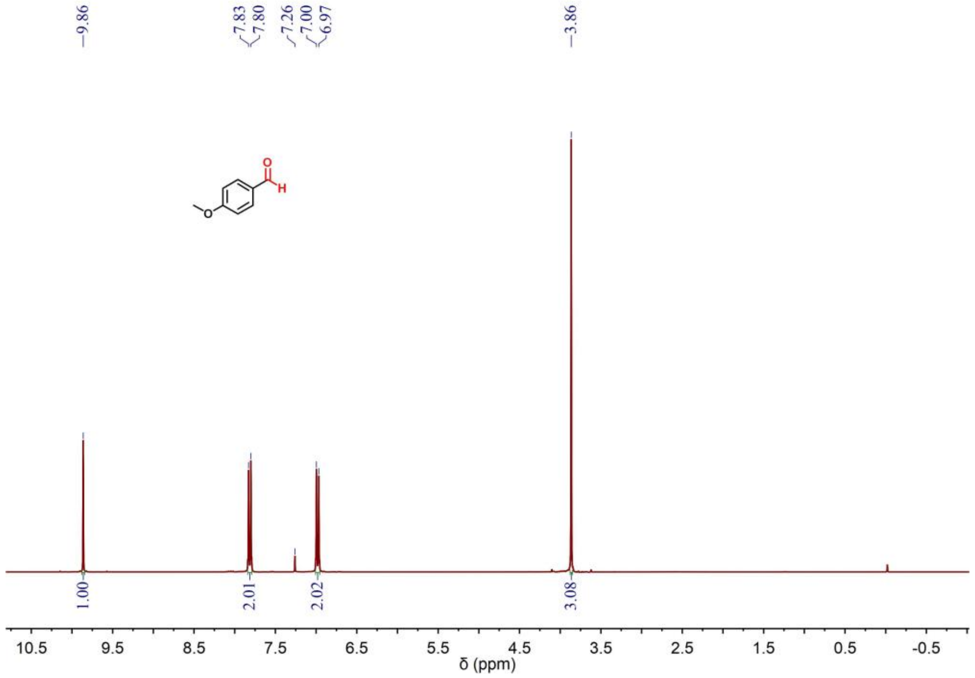

Fig. S25 ^{1}H NMR spectrum (300 MHz, $CDCl_3$, 298 K) of **2e**.

2f: 2-naphthaldehyde, light yellow solid, 91% yield, ^{1}H NMR (300 MHz, $CDCl_3$, 298 K) : δ (ppm) = 10.17 (s, 1H, -C*H*O), 8.35 (s, 1H, Ar-*H*), 8.03 ~ 7.90 (m, 4H, Ar-*H*), 7.67 ~ 7.57 (m, 2H, Ar-*H*).

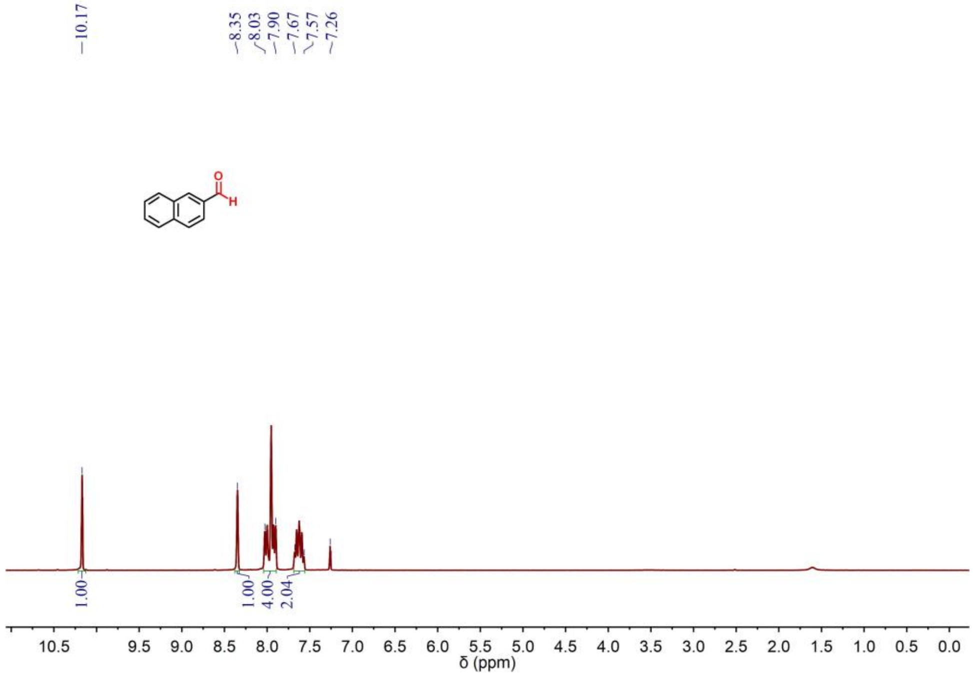

Fig. S26 ^{1}H NMR spectrum (300 MHz, $CDCl_3$, 298 K) of **2f**.

2g: 4-bromobenzaldehyde, white solid, 82% yield, ^{1}H NMR (300 MHz, $CDCl_3$, 298 K) : δ (ppm) = 9.98 (s, 1H, -C*H*O), 7.82 (d, J = 12.0 Hz, 2H, Ar-*H*), 7.51 (d, J = 12.0 Hz, 2H, Ar-*H*).

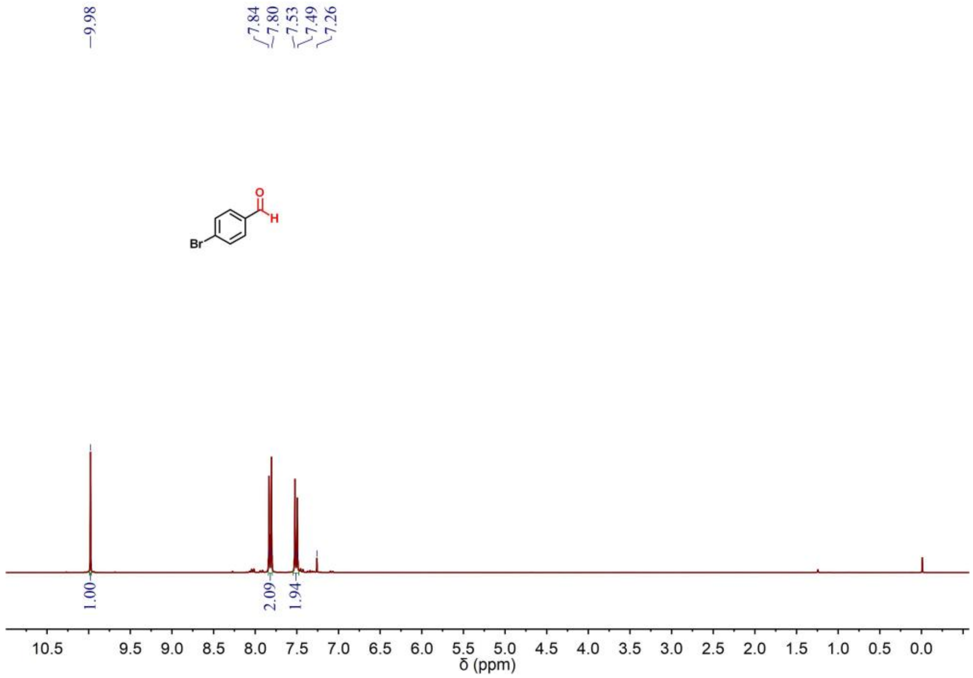

Fig. S27 ^{1}H NMR spectrum (300 MHz, $CDCl_3$, 298 K) of **2g**.

2h: 3-bromobenzaldehyde, pale yellow oily liquid, 84% yield, ^{1}H NMR (300 MHz, $CDCl_3$, 298 K) : *δ* (ppm) = 9.94 (s, 1H, -C*H*O), 7.98 (s, 1H, Ar-*H*), 7.80 ~ 7.71 (m, 2H, Ar-*H*), 7.40 (t, *J* = 7.5 Hz, 1H, Ar-*H*).

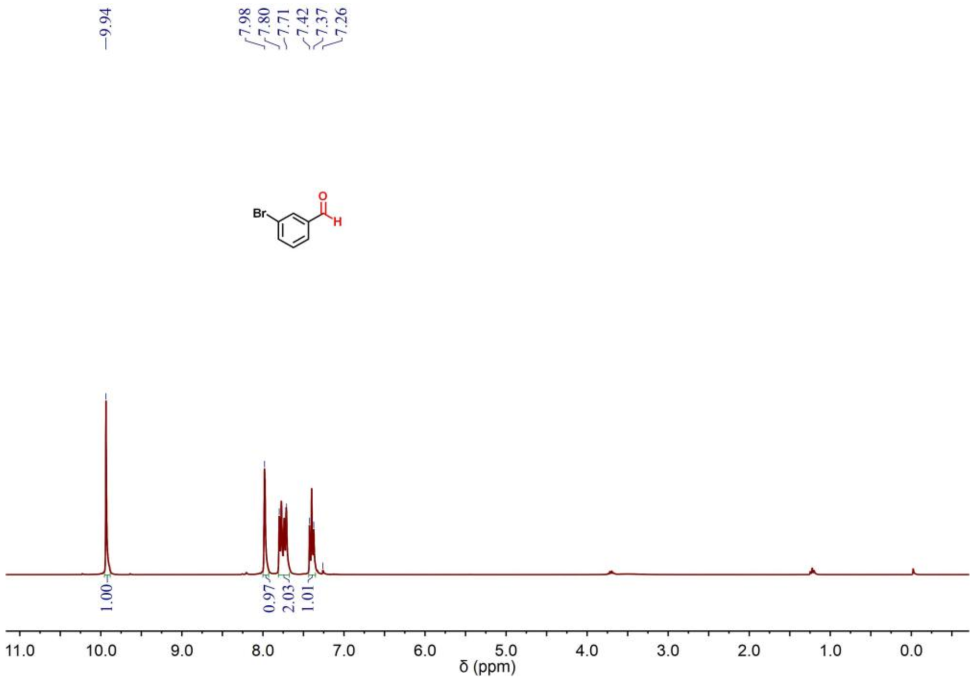

Fig. S28 ^{1}H NMR spectrum (300 MHz, $CDCl_3$, 298 K) of **2h**.

2i: 4-formylbenzoic acid, white solid, 76% yield, ^{1}H NMR (300 MHz, $CDCl_3$, 298 K) : *δ* (ppm) = 10.13 (s, 1H, -C*H*O), 8.27 (d, *J* = 6.0 Hz, 2H, Ar-*H*), 8.00 (d, *J* = 9.0 Hz, 2H, Ar-*H*).

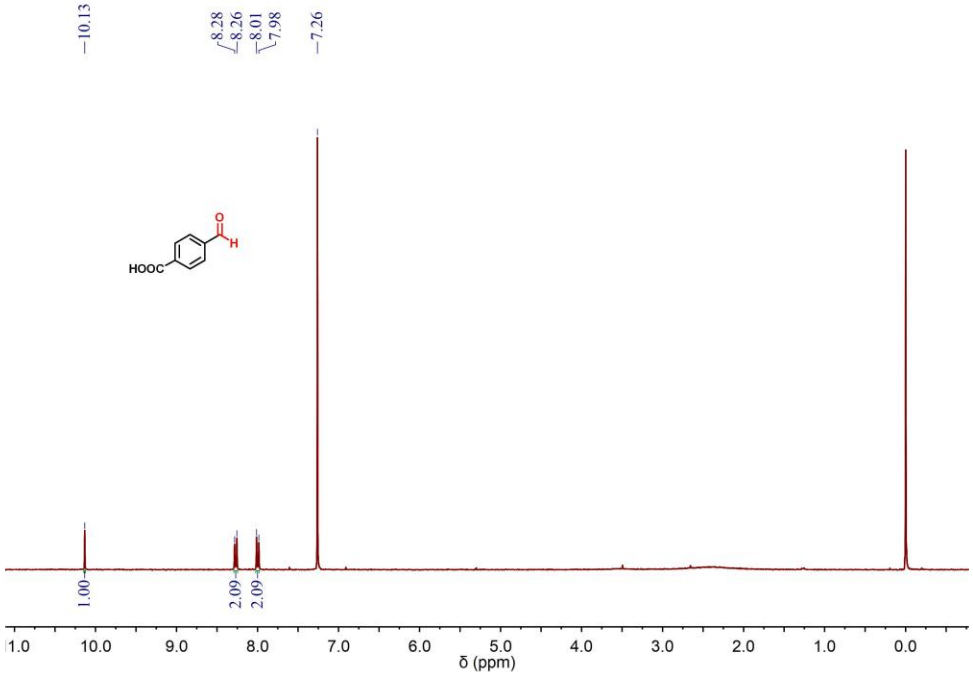

Fig. S29 ^{1}H NMR spectrum (300 MHz, $CDCl_3$, 298 K) of **2i**.

2j: 4-nitrobenzaldehyde, white solid, 71% yield, ^{1}H NMR (300 MHz, $CDCl_3$, 298 K) : δ (ppm) = 10.16 (s, 1H, -C*H*O), 8.40 (d, *J* = 9.0 Hz, 2H, Ar-*H*), 8.08 (d, *J* = 9.0 Hz, 2H, Ar-*H*).

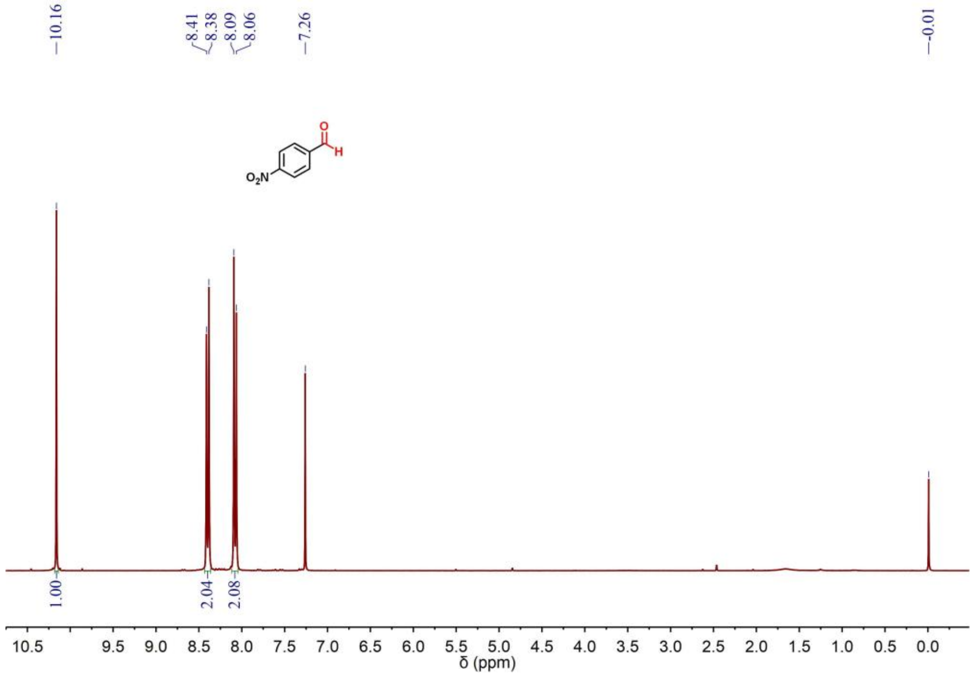

Fig. S30 ^{1}H NMR spectrum (300 MHz, $CDCl_3$, 298 K) of **2j**.

8.3 Photocatalytic activity after numerical heating/cooling cycles

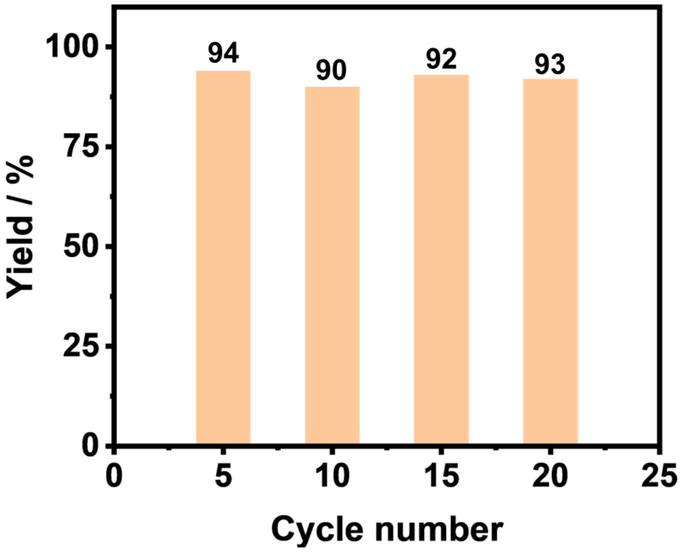

Fig. S31 Bar chart illustrating the photocatalytic efficiency after 5, 10, 15, and 20 heating/cooling cycles between 25 °C and 60 °C.

8.4 Proposed reaction mechanism

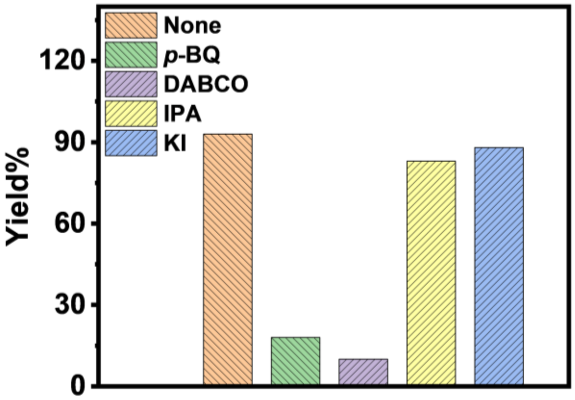

Fig. S32 Control experiments on the photocatalytic oxidative cleavage of 1,1-diphenylethylene in the presence of different reactive oxygen scavengers (*p*-BQ, DABCO, IPA, KI).

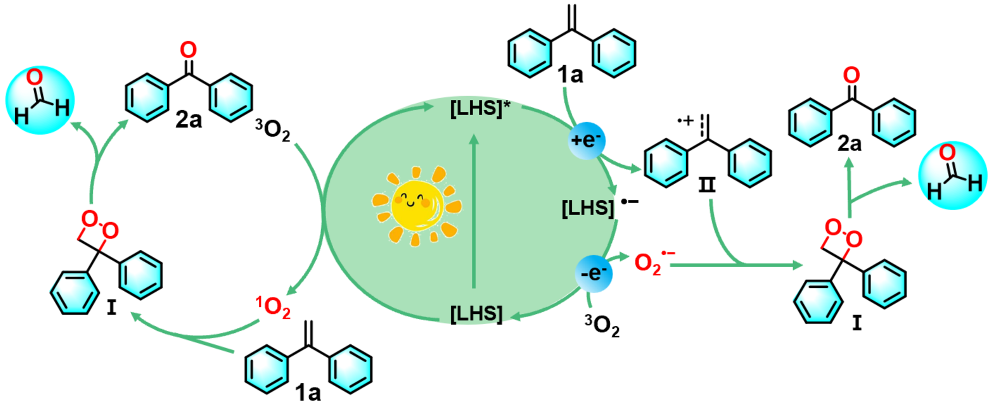

Fig. S33 Proposed reaction mechanism for the photocatalytic oxidative cleavage of 1,1-diphenylethylene, involving different reactive oxygen species ($^1O_2$ on the left and $O_2^{\bullet-}$ on the right).

9. References

[S1] M. D. Tzirakis, M. N. Alberti, H. Weissman, B. Rybtchinski, F. Diederich, *Chem. Eur. J.* **2014**, *20*, 16070.

[S2] L. Wang, R. Zhang, Z. Huang, S. Guo, J. Yang, L. Kong, *Dyes Pigments* **2022**, *197*, 109909.

[S3] M. Hao, G. Sun, M. Zuo, Z. Xu, Y. Chen, X. Y. Hu, L. Wang, *Angew. Chem. Int. Ed.* **2020**, *59*, 10095.

[S4] L. Chen, S.-R. Yan, W.-J. Guo, L. Qiao, X.-Y. Zhan, B. Liu, H.-Q. Peng. *Chem. Sci.* **2024**, *15*, 16059-16068.

[S5] S. Yu, R. X. Zhu, K. Niu, N. Han, H. Liu and L.-B. Xing. *Chem. Sci.* **2024**, *15*, 1870.
